# Diversity of *Cantharellus* (Cantharellales, Basidiomycota) in China with Description of Some New Species and New Records

**DOI:** 10.3390/jof8050483

**Published:** 2022-05-06

**Authors:** Ming Zhang, Chao-Qun Wang, Man-Shui Gan, Yi Li, Shi-Cheng Shao, Wei-Qiang Qin, Wang-Qiu Deng, Tai-Hui Li

**Affiliations:** 1 State Key Laboratory of Applied Microbiology Southern China, Guangdong Provincial Key Laboratory of Microbial Culture Collection and Application, Institute of Microbiology, Guangdong Academy of Sciences, Guangzhou 510070, China; zhangming@gdim.cn (M.Z.); wangcq@gdim.cn (C.-Q.W.); dengwq@gdim.cn (W.-Q.D.); 2 Bureau of Environmental Protection of Dinghai District, Zhoushan Municipality, Zhoushan 316000, China; ganmanshui2022@163.com; 3 School of Food Science and Engineering, Yangzhou University, Yangzhou 225127, China; liyi062@yzu.edu.cn; 4 CAS Key Laboratory of Tropical Plant Resources and Sustainable Use, Xishuangbanna Tropical Botanical Garden, Chinese Academy of Sciences, Mengla 666303, China; shaoshicheng@xtbg.org.cn; 5 School of Marxism, Jishou University, Zhangjiajie 427000, China; qinweiq@163.com

**Keywords:** chanterelle, East Asia, new species, phylogeny, taxonomy

## Abstract

*Cantharellus* is a well-known genus of edible mushrooms, belonging to the family Hydnaceae in the class Agaricomycetes. In this study, a phylogenetic overview of *Cantharellus* subg. *Cinnabarinus* and C. subg. *Parvocantharellus* in China is carried out with the description of four new species. Species description are based on morphological characters of basidiomata and phylogenetic analyses of multi-locus dataset of 28S + *tef*1 + *rpb*2. Among the new species, two species, *C. chrysanthus* and *C. sinocinnabarinus,* belong to *C.* subg. *Cinnabarinus* and two new species, *C. convexus* and *C. neopersicinus*, belong to *C.* subg. *Parvocantharellus*. Species delimitation characters of the new taxa are compared with closely related species. In addition, three new records of *Cantharellus* are reported for China: *C. albovenosus* and *C. citrinus* of subg. *Cinnabarinus* and *C. koreanus* of subg. *Parvocantharellus*. A key to the species of subg. *Cinnabarinus* in China was provided.

## 1. Introduction

*Cantharellus* Fr. was firstly described by Fries [1] based on the type species *Cantharellus cibarius* Fr. Most *Cantharellus* species are popular edible mushrooms, especially beloved in Europe. *Cantharellus* is an ectomycorrhizal genus, forming symbiosis with various plants, such as the trees of Fagaceae, Pinaceae, Betulaceae, Salicaceae, Juglandaceae, Leguminosae, etc. [2,3,4,5,6,7,8]. Species in *Cantharellus* are widely distributed and are especially rich in subtropical to tropical zones [3,9,10]. Up to now, about 300 species of *Cantharellus* have been reported worldwide [7]. However, the species diversity is poorly known in Asia in the past decades, and many specimens were named after European or North American species [6,11,12,13]. In recent years, some new species were reported from Asia based on the combination of morphological characters and DNA phylogenetic analyses [6,7,11,12,13,14,15,16]. Recent phylogenetic studies demonstrated that *Cantharellus* is monophyletic and forms a sister relationship with *Craterellus* Pers. [3,4,7]. Species in *Cantharellus* were divided into seven subgenera based on multi-locus phylogenetic analyses in Buyck et al. [3], and a subsequent study in Cao et al. [7]. *Cantharellus* subg. *Cinnabarinus* Buyck & V. Hofst., typified by *C. cinnabarinus* (Schwein.) Schwein. was introduced for a monophyletic assemblage of mostly quite small, yellow, orange, pink or red species, sometimes mixed with lilac-purple or brownish tones in the pileus center, strongly veined in the lamellate hymenophore with principally thin-walled hyphal endings and abundant in clamp connections [3,17,18]. Species in subg. *Cinnabarinus* are widely distributed in Asia, Europe, North America, Australasia and Africa, and 16 species have been reported worldwide. In China, a large number of *Cantharellus* species have been reported, but only two species in the subg. *Cinnabarinus* were recorded, i.e., *C. cinnabarinus* and *C. phloginus,* by S.C. Shao & P.G. Liu. *Cantharellus cinnabarinus*, originally reported from North America, was recorded to be widely distributed in China [19,20,21]; *C. phloginus* was described as being from southwestern China [22].

In this study, a number of *Cantharellus* specimens were collected from China; further study proved that they represented eight distinct species, five of which belong to the subg. *Cinnabarinus* and three to the subg. *Parvocantharellus*. Four species are described below as new to science, which would make a contribution to understanding the species diversity of *Cantharellus* in China, and revealing the phylogenetic relationships of *Cantharellus* species.

## 2. Materials and Methods

### 2.1. Morphological Studies

Photographs of fresh basidiomata were taken in the field. Specimens were dried and deposited in the Fungarium of Guangdong Institute of Microbiology (GDGM). Descriptions of macro-morphological characters and habitats were obtained from photographs and field notes. The color codes followed Kornerup and Wanscher [23]. Microscopic observations were carried out on tissue sections stained with 5% aqueous KOH and 1% aqueous Congo red under a light microscope (Carl Zeiss Microscopy GmbH, Göttingen, Germany) with a magnification up to 1000×. For basidiospore descriptions, the notation (a–)b–c(–d) describes basidiospore dimensions, where the range b–c represented 90% or more of the measured values and ‘a’ and ‘d’ were the extreme values; L_m_ and W_m_ indicated the average length and width (±standard deviation) of the measured basidiospores, respectively; Q referred to the length/width ratio of an individual basidiospore and Q_m_ referred to the average Q value of all basidiospores ± sample standard deviation. All line-drawings of microstructures were made based on rehydrated materials.

### 2.2. DNA Extraction, PCR Amplification and Sequencing

Genomic DNA was extracted from the voucher specimens using the Sangon Fungus Genomic DNA Extraction kit (Sangon Biotech Co., Ltd., Shanghai, China) according to the manufacturer’s instructions. Primer pairs LROR/LR7 [24], tef1F/tef1R and RPB2-5FCanth/RPB2-7cRCanth [3,25] were used to amplify the LSU, *tef1* and *rpb2* region, respectively. PCR reactions were performed in a total volume of 25 μL containing 0.5 μL template DNA, 11 μL sterile deionized water, 0.5 μL of each primer and 12.5 μL 2 × PCR mix [DreamTaqtm Green PCR Master Mix (2×), Fermentas, USA]. Amplification reactions were performed in a Tprofessional Standard thermocycler (Biometra, Göttingen, Germany) under the following conditions: 95 °C for 4 min; then, 35 cycles of denaturation at 94 °C for 60 s, annealing at 53 °C (LSU)/50 °C (*tef*1)/52 °C (*rpb*2) for 60 s and extension at 72 °C for 60 s; with a final extension at 72 °C for 8 min. The PCR products were electrophoresed on 1% agarose gels and then send for sequencing on an ABI Prism^®^ 3730 Genetic Analyzer (PE Applied Biosystems, Foster, CA, USA) at the Beijing Genomic Institute (BGI) using the same PCR primers. The raw sequences were assembled and checked with SeqMan implemented in Lasergene v7.1 (DNASTAR Inc., Madison, WI, USA). The newly generated sequences in this study were submitted to GenBank.

### 2.3. Phylogenetic Analyses

Sequences generated in this study and those downloaded from GenBank were combined and used for phylogenetic reconstruction. Detailed information of specimens included in this study was given in Table 1. Three sequence matrices, i.e., nrLSU, *tef1* and *rpb2*, were aligned separately with software MAFFT v6.853 using the E-INS-i strategy [26] and then manually adjusted in MEGA 6 [27]. The ambiguous aligned regions and introns of the two protein-coding genes of *tef1* and *rpb2* were retained in the final analyses.

Maximum Likelihood (ML) analyses were inferred using RAxML v7.2.6 [28], and all parameters were kept as defaults except for choosing GTRGAMMAI as the model; statistical supports were obtained using rapid non-parametric bootstrapping with 1000 replicates. Bayesian Inference (BI) phylogenies were inferred using MrBayes 3.2.6 [29]; the best models of the multi-locus datasets were searched via the PartitionFinder 2 [30] for each locus, i.e., K80 + I + G, SYM + I + G and SYM + I + G for 28S, *tef1* and *rpb2*, respectively. BI analysis using 4 chains were conducted by setting generations to 20 million and stoprul command with the value of stopval set to 0.01; trees were sampled every 1000 generations, the first 25% generations were discarded as burn-in and posterior probabilities (PP) were then calculated from the posterior distribution of the retained Bayesian trees. *Cantharellus cibarius* Fr. was selected as the outgroup based on recent studies [3,13]. The phylogenetic trees were visualized using FigTree v1.4.23.

**Table 1:** Specimen information used in this study. Sequences newly generated in this study are in bold; HT, NT and ET refer to holotype, neotype and epitype, respectively.

Taxa	Voucher	Locality	GenBank Accession No.			Reference
			LSU	*tef*1	*rpb*2	
*Cantharellus afrocibarius*	BB 96.236	Zambia	KF294669	JX192994	KF294747	[3]
*C. afrocibarius*	BB 96.235 (HT)	Zambia	KF294668	JX192993	KF294746	[3]
*C. albovenosus*	1690 (HT)	South Korean	–	KY271942	–	[11]
*C. albovenosus*	1713	South Korean	–	MW124387	–	[11]
***C. albovenosus***	**GDGM85853**	**China**	**OM978952**	**ON119062**	**ON119006**	**Present study**
***C. albovenosus***	**GDGM85846**	**China**	**OM978950**	**ON119060**	**ON119004**	**Present study**
***C. albovenosus***	**GDGM85142**	**China**	**OM978949**	**ON119059**	**ON229082**	**Present study**
***C. albovenosus***	**HMAS279296**	**China**	**OM978948**	**ON119066**	**ON119010**	**Present study**
***C. albovenosus***	**HMAS279284**	**China**	**ON212414**	**ON119064**	**ON119008**	**Present study**
***C. albovenosus***	**HMAS279292**	**China**	**ON212412**	**ON119065**	**ON119009**	**Present study**
***C. albovenosus***	**HMAS279262**	**China**	**OM978947**	**ON119063**	**ON119007**	**Present study**
***C. albovenosus***	**GDGM85852**	**China**	**OM978951**	**ON119061**	**ON119005**	**Present study**
*C. albus*	HKAS107045 (HT)	China	MT782540	MT776015	MT776012	[12]
*C. albus*	GDGM81399	China	MZ605074	MZ613977	MZ614022	[13]
*C. albus*	GDGM81064	China	MZ605073	MZ613976	MZ614021	[13]
*C. appalachiensis*	GRSM77088	USA	DQ898690	–	DQ898748	[31]
*C. appalachiensis*	BB 07.123	USA	KF294635	GQ914979	KF294711	[3]
*C. aurantinus*	GDGM46278 (HT)	China	MZ766517	MZ766560		[13]
*C. aurantinus*	GDGM46279	China	MZ766518	MZ766561	MZ766571	[13]
*C. aurantinus*	GDGM81899	China	MZ766520	MZ766563	MZ766573	[13]
*C. aurantinus*	GDGM84974	China	MZ766521	MZ766564	MZ766572	[13]
*C. austrosinensis*	GDGM81303	China	MZ605084	MZ613986	MZ614029	[13]
*C. austrosinensis*	GDGM81249 (HT)	China	MZ605082	MZ613983	MZ614027	[13]
*C. austrosinensis*	GDGM80616	China	MZ605081	MZ613982	MZ614026	[13]
*C. austrosinensis*	GDGM81381	China	MZ605086	MZ613988	MZ614031	[13]
*C. austrosinensis*	GDGM81379	China	MZ605085	MZ613987	MZ614030	[13]
*C. austrosinensis*	GDGM81985	China	MZ605087	MZ613989	MZ614032	[13]
***C. chrysanthus***	**GDGM45166**	**China**	**OM978959**	**ON119074**	**ON119011**	**Present study**
***C. chrysanthus***	**GDGM45937**	**China**	**OM978960**	**ON119075**	**ON119012**	**Present study**
***C. chrysanthus***	**GDGM85298**	**China**	**OM978975**	**ON119089**	**ON119025**	**Present study**
***C. chrysanthus***	**GDGM85305**	**China**	**OM978976**	**ON119090**	**ON119026**	**Present study**
***C. chrysanthus***	**GDGM53485**	**China**	**OM978962**	**ON119077**	**ON119014**	**Present study**
***C. chrysanthus***	**GDGM80220 (HT)**	**China**	**OM978970**	**ON119083**	**ON119019**	**Present study**
***C. chrysanthus***	**GDGM82511**	**China**	**OM978973**	**ON119087**	**ON119023**	**Present study**
***C. chrysanthus***	**GDGM82516**	**China**	**OM978974**	**ON119088**	**ON119024**	**Present study**
***C. chrysanthus***	**GDGM80436**	**China**	**OM978971**	**ON119084**	**ON119020**	**Present study**
***C. chrysanthus***	**GDGM80202**	**China**	**OM978965**	**ON119080**	**ON119016**	**Present study**
***C. chrysanthus***	**GDGM80204**	**China**	**OM978966**	**ON119081**	**ON119017**	**Present study**
***C. chrysanthus***	**HMAS279434**	**China**	**ON212413**	**ON119091**	**ON229079**	**Present study**
***C. chrysanthus***	**GDGM80438**	**China**	**–**	**ON119085**	**ON119021**	**Present study**
***C. chrysanthus***	**GDGM82473**	**China**	**OM978972**	**ON119086**	**ON119022**	**Present study**
***C. chrysanthus***	**GDGM77035**	**China**	**OM978964**	**ON119079**	**ON229081**	**Present study**
***C. chrysanthus***	**GDGM60524**	**China**	**OM978963**	**ON119078**	**ON119015**	**Present study**
***C. chrysanthus***	**GDGM80217**	**China**	**OM978969**	**ON119082**	**ON119018**	**Present study**
***C. chrysanthus***	**GDGM49628**	**China**	**OM978961**	**ON119076**	**ON119013**	**Present study**
***C. chrysanthus***	**GDGM87950**	**China**	**OM978968**	**–**	**ON119027**	**Present study**
***C. chrysanthus***	**GDGM87951**	**China**	**OM978967**	**–**	**ON119028**	**Present study**
*C. cibarius*	GE 07.025	France	KF294658	GQ914949	KF294736	[3]
*C. cibarius*	BB 07.300	Slovakia	KF294641	GQ914950	KF294718	[3]
*C. cinnabarinus*	BB 04.263 (NT)	USA	–	GQ914983	–	[32]
*C. cinnabarinus*	BB 07.053	USA	KF294630	GQ914984	KF294705	[32]
*C. cinnabarinus*	BB 07.001	USA	KF294624	GQ914985	KF294698	[32]
*C. citrinus*	1691 (HT)	South Korean	–	MW124385	–	[16]
*C. citrinus*	1715	South Korean	–	MW124388	–	[16]
*C. citrinus*	1710	South Korean	–	MW124386	–	[16]
*C. citrinus*	1711	South Korean	–	MW124384	–	[16]
***C. citrinus***	**GDGM86140**	**China**	**OM978955**	**ON119070**	**ON119032**	**Present study**
***C. citrinus***	**GDGM86141**	**China**	**OM978956**	**ON119071**	**ON119033**	**Present study**
***C. citrinus***	**GDGM80825**	**China**	**–**	**ON119069**	**ON119031**	**Present study**
***C. citrinus***	**GDGM86142**	**China**	**OM978957**	**ON119072**	**ON119034**	**Present study**
***C. citrinus***	**GDGM80724**	**China**	**OM978954**	**ON119068**	**ON119030**	**Present study**
***C. citrinus***	**GDGM86143**	**China**	**OM978958**	**ON119073**	**ON119035**	**Present study**
***C. citrinus***	**GDGM80723**	**China**	**OM978953**	**ON119067**	**ON119029**	**Present study**
*C. coccolobae*	1064_RC. 14_24	Guadeloupe	KX857088	KX857020	KX856992	[33]
*C. coccolobae*	1065_RC. 11_25 (HT)	Guadeloupe	KX857089	KX857021	KX856993	[33]
*C. congolensis*	1645/BB16.044	Saharan Africa	KX857102	KX857075	KX857006	[33]
*C. congolensis*	1676/BB16.123	Saharan Africa	KX857106	KX857078	KX857010	[33]
*C.* aff. *congolensis*	BB 06.176	Madagascar	KF294606	–	KF294680	[3]
*C.* aff. *congolensis*	BB 06.197	Madagascar	KF294608	–	KF294683	[3]
***C. convexus***	**GDGM54841**	**China**	**OM978940**	**ON119052**	**ON119036**	**Present study**
***C. convexus***	**GDGM70307 (HT)**	**China**	**OM978941**	**ON119053**	**ON119037**	**Present study**
*C. corallinus*	1083_JJ_MO_CANT_2	USA	–	KX857031	–	[34]
*C. corallinus*	1086_JJ_MO_CANT_5	USA	–	KX857034	–	[34]
*C. corallinus*	FLAS_F_61106	USA	–	MK045368	–	[34]
*C. curvatus*	BRNM:825749 (HT)	South Korea		MW124390		[16]
*C. cyphelloides*	TNS F-61721 (HT)	Japan	NG059027	–	–	[35]
*C. decolorans*	BB 08.278 (HT)	Madagascar	KF294654	GQ914968	KF294731	[3]
*C. fistulosus*	DT_43	Tanzania	JQ976965	JX192997	–	[3]
*C. friesii*	AH44798	Spain	KR677522	KX828831	KX828752	[36]
*C. friesii*	VDKO 1165	Africa	–	KX834408	KX881922	[5]
*C. galbanus*	GDGM86249 (HT)	China	ZM766516	MZ766568	MZ766577	[13]
*C. garnierii*	BB 09.024	New Caledonia	KX857085	KX857017	KX856989	[34]
*C. garnierii*	BB 09.283	New Caledonia	KX857087	KX857019	KX856991	[34]
*C. garnierii*	BB 09.033	New Caledonia	KX857086	KX857018	KX856990	[34]
*C. garnierii*	RF33	New Caledonia	AY392768	–		[37]
*C. garnierii*	RF32	New Caledonia	AY392767	–		[37]
*C. koreanus*	1697	South Korea	–	KY271940	–	[11]
*C. koreanus*	1689 (HT)	South Korea	–	KY271941	–	[11]
***C. koreanus***	**GDGM85306**	**China**	**OM978978**	**ON119093**	**ON229077**	**Present study**
***C. koreanus***	**GDGM79233**	**China**	**OM978977**	**ON119092**	**ON229078**	**Present study**
*C. koreanus*	1693	South Korea	–	–		Unpublished
*C. koreanus*	1694	South Korea	–	–		Unpublished
*C. koreanus*	1696	South Korea	–	–		Unpublished
*C. luteolus*	GDGM60393 (HT)	China	ZM766515	MZ766566	MZ766575	[13]
*C. luteolus*	GDGM86247	China	MZ766513	MZ766567	MZ766576	[13]
*C. luteolus*	GDGM44258	China	ZM766514	MZ766566	MZ766570	[13]
*C. luteovirens*	GDGM81079	China	MZ605092	MZ613994	MZ614036	[13]
*C. luteovirens*	GDGM80672 (HT)	China	MZ605090	MZ613992	MZ614035	[13]
*C. luteovirens*	GDGM80680	China	MZ605091	MZ613993	–	[13]
*C. minioalbus*	GDGM78910	China	MZ605098	MZ613999	MZ614043	[13]
*C. minioalbus*	GDGM78901 (HT)	China	MZ605097	MZ613998	MZ614042	[13]
*C. minioalbus*	GDGM78916	China	MZ605100	MZ614001	MZ614045	[13]
*C. minor*	BB 07.057	USA	KF294632	JX192979	KF294707	[3]
*C. minor*	BB 07.002	USA	KF294625	JX192978	KF294699	[3]
***C. neopersicinus***	**GDGM85145-1**	**China**	**OM978942**	**ON119054**	**ON119039**	**Present study**
***C. neopersicinus***	**GDGM85145-2**	**China**	**OM978945**	**ON119055**	**ON119040**	**Present study**
***C. neopersicinus***	**GDGM85145-3**	**China**	**OM978946**	**ON119056**	**ON119041**	**Present study**
***C. neopersicinus***	**GDGM87366-1 (HT)**	**China**	**OM978943**	**ON119057**	**ON119042**	**Present study**
***C. neopersicinus***	**GDGM87366-2**	**China**	**OM978944**	**ON119058**	**ON119043**	**Present study**
***C. phloginus***	**GDGM79007-1**	**China**	**OM978979**	**ON119094**	**ON119044**	**Present study**
***C. phloginus***	**GDGM79007-2**	**China**	**OM978980**	**ON119095**	**ON119045**	**Present study**
*C. phloginus*	SSC99 (HT)	China	–	KF801096	–	[22]
*C. phloginus*	SSC98	China	–	KF801095	–	[22]
*C. phloginus*	Yuan14468	China	–	MW999424.	–	[7]
*C. phloginus*	Yuan14490	China	–	MW999425	–	[7]
***C. phloginus***	**GDGM82514**	**China**	**–**	**ON119096**	**–**	**Present study**
*C. pseudominimus*	JV 00.663	Portugal	KF294657	JX192991	KF294735	[3,10]
*C. romagnesianus*	AH44218	Spain	KX828807	KX828836	KX828757	[36]
*C. roseofagetorum*	AH44789	Georgia	KX828812	KX828839	KX828760	[36]
***C. sinocinnabarinus***	**GDGM83229**	**China**	**OM978983**	**ON119098**	**ON119047**	**Present study**
***C. sinocinnabarinus***	**GDGM83238**	**China**	**OM978985**	**ON119101**	**ON119051**	**Present study**
***C. sinocinnabarinus***	**GDGM83023**	**China**	**OM978981**	**ON119097**	**ON119050**	**Present study**
***C. sinocinnabarinus***	**GDGM83232**	**China**	**–**	**ON119100**	**ON119049**	**Present study**
***C. sinocinnabarinus***	**GDGM83027**	**China**	**OM978982**	**–**	**ON119046**	**Present study**
***C. sinocinnabarinus***	**GDGM83230 (HT)**	**China**	**OM978984**	**ON119099**	**ON119048**	**Present study**
*C. sinocinnabarinus*	HKAS58243	China	JF906727	–	–	[20]
*C. sinominor*	GDGM80788	China	MZ605105	MZ614004	MZ614048	[13]
*C. sinominor*	GDGM80842 (HT)	China	MZ605107	MZ614006	MZ614050	[13]
*C. sinominor*	GDGM80885	China	MZ605108	MZ614007	MZ614051	[13]
*C. aff. subcyanoxanthus*	BB 98.014	Tanzania	KF294615	JX192973	KF294689	[3]
*C. tabernensis*	BB 07.119	USA	KF294634	GQ914976	KF294709	[3]
*C. tabernensis*	BB 07.056 (ET)	USA	KF294631	GQ914974	KF294706	[3,38]
*C. texensis*	341/O7.120	USA	JN940601	GQ914987	KF294710	[3]
*C. texensis*	BB 07.018	USA	KF294626	GQ914988	KF294701	[3]
*C. xanthocyaneus*	1751	Congo	MT006309	MT002277	–	[39]
*C. xanthocyaneus*		Congo	MT006310	MT002278	–	[39]
*C. zangii*	GDGM82389	China	MZ605110	MZ614009	MZ614053	[13]
*C. zangii*	GDGM82393	China	MZ605111	MZ614010	MZ614054	[13]
*C. zangii*	GDGM82374	China	MZ605109	MZ614008	MZ614052	[13]

## 3. Results

### 3.1. Molecular Phylogeny

**Figure 1:**
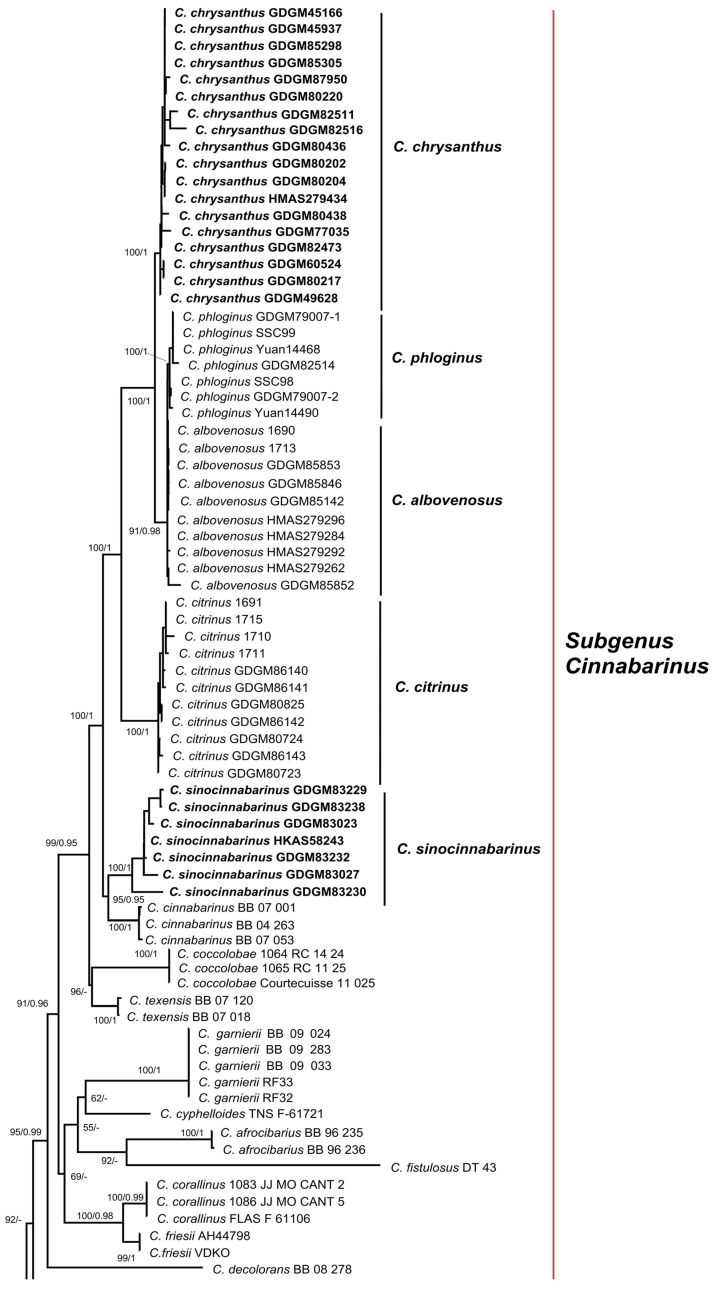

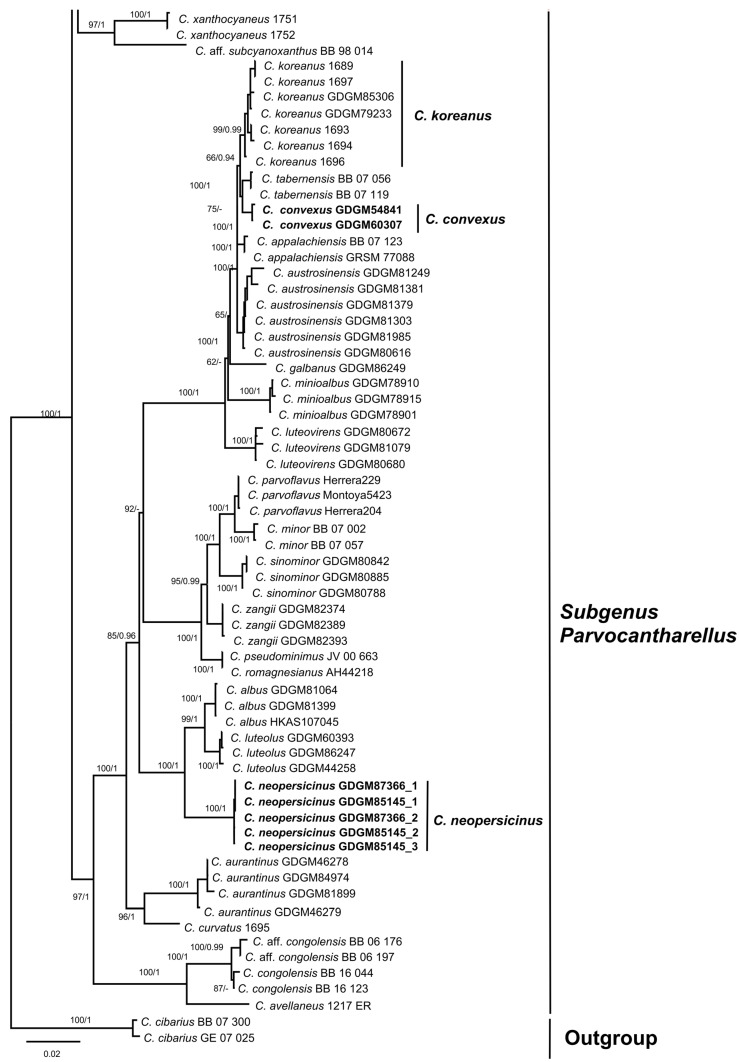
Phylogenetic tree of representative species of *Cantharellus* inferred from LSU-*tef1*-*rpb2* dataset by means of both ML and BI methods. *Cantharellus cibarius* Fr. served as outgroup. Bootstrap Supports (BS > 50%) and Bayesian Posterior Probabilities (BPP > 0.90) are shown on the supported branches. Bold names represent new species.

For phylogenetic analyses, a total of 152 sequences were newly produced in this study, containing 49 nrLSU, 51 *tef1* and 52 *rpb2*, and 185 reliable sequences were downloaded from the GenBank database based on previous studies [3,13]. The combined dataset (LSU + *tef1* + *rpb2*) contained 2892 characters (1311, 707 and 874 for LSU, *tef1* and *rpb2,* respectively), of which 2013 were conserved and 708 were parsimony-informative. ML and BI analyses of the concatenated data set resulted in almost identical topologies, and no strongly-supported conflicts between ML and BI analyses were discovered; thus, only the tree inferred from ML analysis was displayed (Figure 1). Our phylogenetic analyses indicated that members of *C.* subg. *Cinnabarinus* formed a highly support monophyletic group (MLB/BPP = 100%/1.0). Five well-supported clades in the subg. *Cinnabarinus* were identified based on samples newly collected from China, including two new species, two species newly recorded in China and a known species in China. Besides, three well-supported clades in the subg. *Parvocantharellus* were firstly discovered in China, containing two new species and a newly recorded species from China.

### 3.2. Taxonomy

#### 3.2.1. *Cantharellus* subgen. *Cinnabarinus* Buyck & V. Hofst.

**Figure 2:**
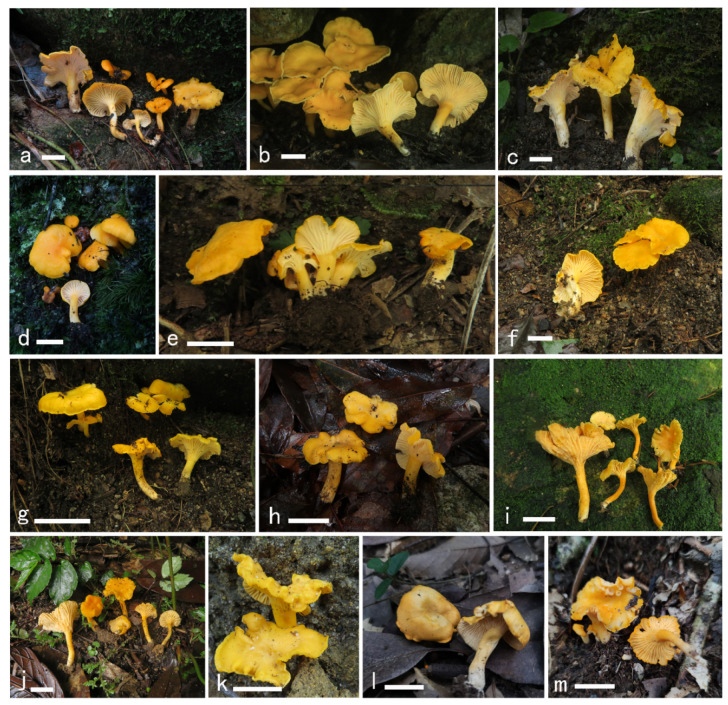
Basidiomata of *Cantharellus chrysanthus.* (**a**,**b**) GDGM80220, holotype. (**c**) GDGM60524. (**d**) GDGM80438. (**e**) GDGM82516. (**f**) GDGM80217. (**g**) GDGM49628. (**h**) GDGM80436. (**i**) GDGM60334. (**j**) GDGM80202. (**k**) GDGM45937. (**l**) GDGM85298. (**m**) GDGM82473. Bars = 2 cm.

**Figure 3:**
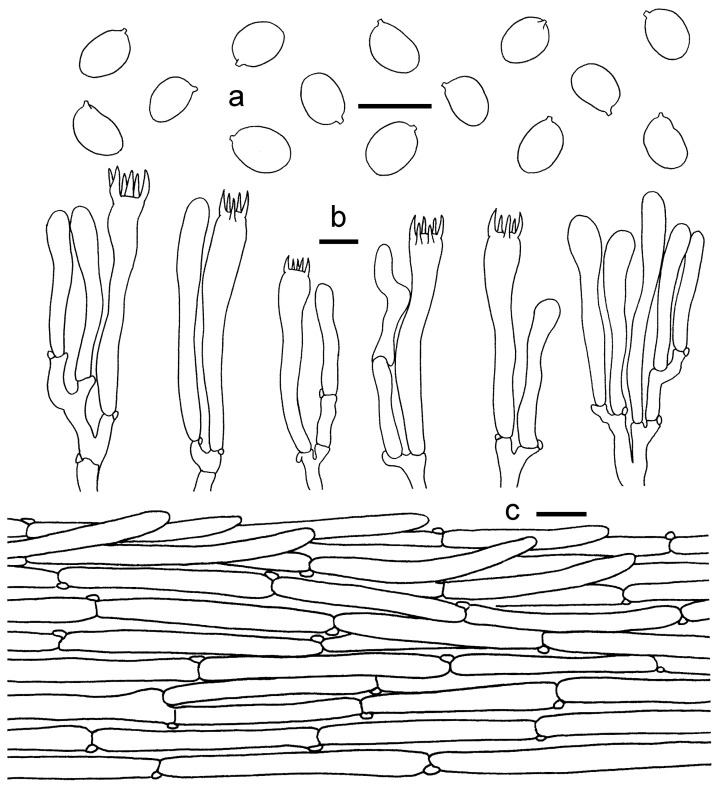
*Cantharelluschrysanthus* (GDGM80220, Holotype). (**a**) Basidiospores. (**b**) Basidia, basidiola and elements of the subhymenium. (**c**) Pileipellis. Bars: (**a**,**b**) = 10 μm; (**c**) = 20 μm.

***Cantharellus chrysanthus*** Ming Zhang, C.Q. Wang & T.H. Li sp. nov.; Figure 2 and Figure 3.

MycoBank: MB843657.

GenBank: OM978970 for LSU, ON119083 for *tef1* and ON119019 for *rpb2*.

Etymology—refers to the color of pileus similar to the yellow chrysanthemum flower.

Diagnosis—This species is characterized by its orange to orange-yellow pileus, pinkish white to orange white hymenophore, thin-walled pileipellis terminal hyphae, broadly ellipsoid basidiospores (7.5–9 × 5–6.5 μm) and long basidia up to 100 μm.

Type—CHINA. Guangdong Province, Shaoguan City, Ruyuan town, Nanling National Natural Reserve, alt. 500 m, 10 June 2020, Ming Zhang (GDGM80220).

Basidiomata small-sized. Pileus 20–60 mm broad, convex, with involute margin when young, then gradually to nearly applanate or broadly infundibuliform with depressed center and inflexed to straight, irregularly undulate or slightly cracked at maturity; surface dry or hygrophanous, glabrous or finely subtomentose, orange (5A7–6A7) to deep orange (5A8–6A8) when young, slightly fading to orange yellow to yellow (3A7–4A7) when mature. Context yellowish white to orange white (4A2–6A2), 1–2 mm thick in the center of the pileus, sharply attenuate towards margin, unchanging when exposed. Hymenophore decurrent, subdistant, composed of bifurcate, 2–3 mm high venose folds, particularly towards pileus margin, pinkish white (7A2–10A2), but in some specimens yellowish white to orange white, unchanging when bruised. Stipe 20–60 × 3–14 mm, central, cylindrical or slightly tapering towards base, solid, glabrous or finely pubescent, concolorous with pileus or paler, unchanging when handled. Odor fruity and pleasant. Taste mild.

Basidiospores 7.5–9 × 5–6.5 μm, L_m_ × W_m_ = 8.45(±0.47) × 5.98(±0.42) μm, Q = (1.25)1.28–1.6(1.64), Q_m_ = 1.42 ± 0.1; broadly elliptical to subglobose, smooth, guttulate, thin-walled. Basidia 55–100 × 7–11 μm, 2–6-spored, narrowly clavate, colorless to hyaline in KOH; sterigmata 6–10 μm long. Pileipellis a cutis with long, repent and occasionally interwoven hyphae, subcylindrical cells that are 6–12 μm wide, thin-walled. Stipitipellis a cutis of cylindrical, parallel hyphae, 3–8 μm wide. Clamp connections abundant in all tissues.

Habitat and distribution—Solitary or scattered under Fagaceae trees mixed with other broadleaf trees in subtropical forests. Known from southern and southwestern China.

Additional specimen examined—China. Guangdong Province, Shaoguan City, Ruyuan town, Nanling National Natural Reserve, alt. 500 m, 7 June 2017, Ming Zhang (GDGM49628); same location, alt. 500 m, 21 July 2017, Ming Zhang (GDGM60524); same location, alt. 500 m, 9 June 2020, Ming Zhang (GDGM80436, GDGM80438); same location, alt. 500 m, 10 June 2020, Ming Zhang (GDGM80202, GDGM80204, GDGM80217, GDGM80220,); Huizhou city, Xiangtoushan National Natural Reserve, alt. 550 m, 17 May 2016, Ting Li (GDGM45937); Hunan Province, Rucheng town, Jiulongjiang National Forest Park, alt. 300 m, 4 September 2016, Ming Zhang (GDGM53485); Zhejiang province, Jinhua city, Wuyi Town, 23 August 2015, Tai-Hui Li (GDGM45166); Hangzhou City, Laohushan, 15 July 2021, Bao-Juan Ling (GDGM85298, GDGM85305); Qingyuan Town, Baishanzu National Natural Reserve, alt. 29 July 2020, Tai-Hui Li (GDGM82473); Longquan City, Fengyangshan National Natural Reserve, 25 August 2016, Rui-Lin Zhao (ZRL20161616, HMAS279434); Quzhou City, Kaihua County, He Tian township, Chi Keng village, 24 May 2021, Yi Li (GDGM87950); Quzhou City, Kaihua County, Shengtangou Scenic Spot, 30 May 2021, Yi Li (GDGM87951); Anhui Province, Huangshan City, Huangshan scenic spot, 11 August 2020, Ming Zhang (GDGM82511), same location, 13 August 2020, Ming Zhang (GDGM82516); Guizhou Province, Guiyang City, Longli County, Guanyin Village, bought from a wild mushroom market, 2 August 2019, alt. 1000 m, Yong He (GDGM77035).

Notes—*Cantharellus chrysanthus* is different from other *Cantharellus* species by the combined features of the orange to orange-yellow pileus, the pinkish white to orange white hymenophore, the thin-walled terminal hyphae of pileipellis, the broadly ellipsoid basidiospores (7.5–9 × 5–6.5 μm) and the long basidia up to 100 μm.

Phylogenetically, *C. chrysanthus* is related to *C. albovenosus* and *C. phloginus* in the analyses of the multi-locus datasets. However, *C. albovenosus* differs in its orange to reddish orange pileus with tomentoum or fibrilla, white to orange white and better-developed hymenophore, orange to reddish orange stipe, smaller basidiospores (7–8.5 × 5–6 μm) and shorter basidia (48–63 × 7–9 μm) [11]; *Cantharellus phloginus*, reported from southwest China, differs in its pastel red to pastel pink pileus and stipe, pale yellow to light yellow hymenophore, larger basidiospores [6.8–9.5 (–12) × 5–7 μm] and shorter basidia (60–95 × 8–10 μm) [22].

**Figure 4:**
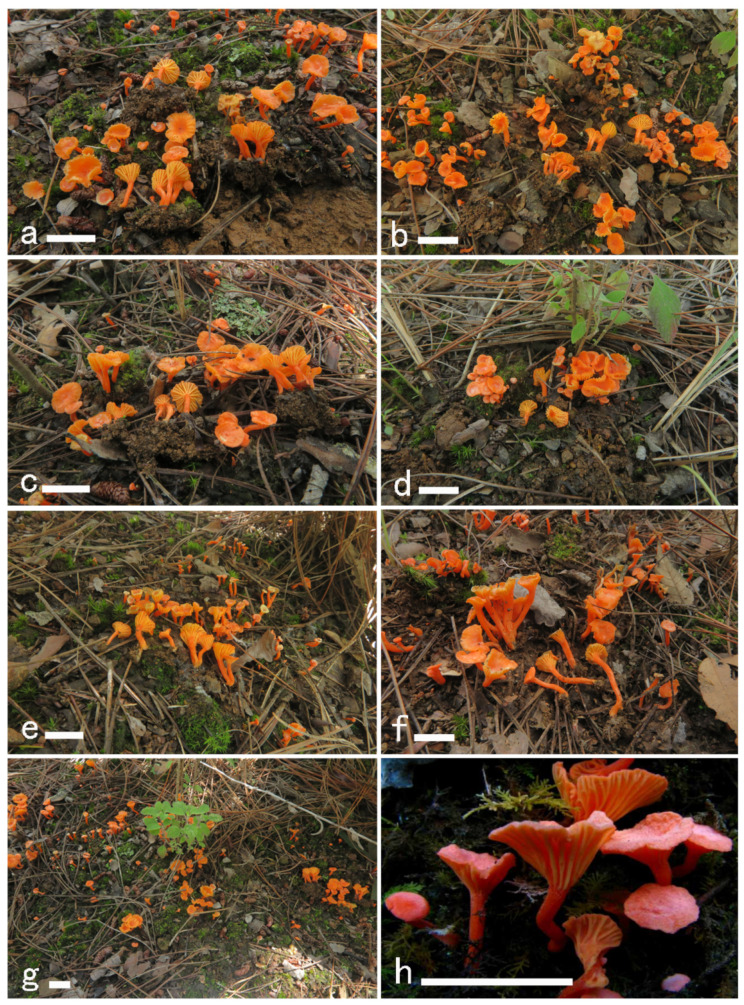
Basidiomata of *Cantharellus sinocinnabarinus.* (**a**,**c**) GDGM83230. (**b**) GDGM83232. (**d**) GDGM83229. (**e**) GDGM832296. (**f**) GDGM83027. (**g**) GDGM83238. (**h**) HKAS58243. Bars = 2 cm.

**Figure 5:**
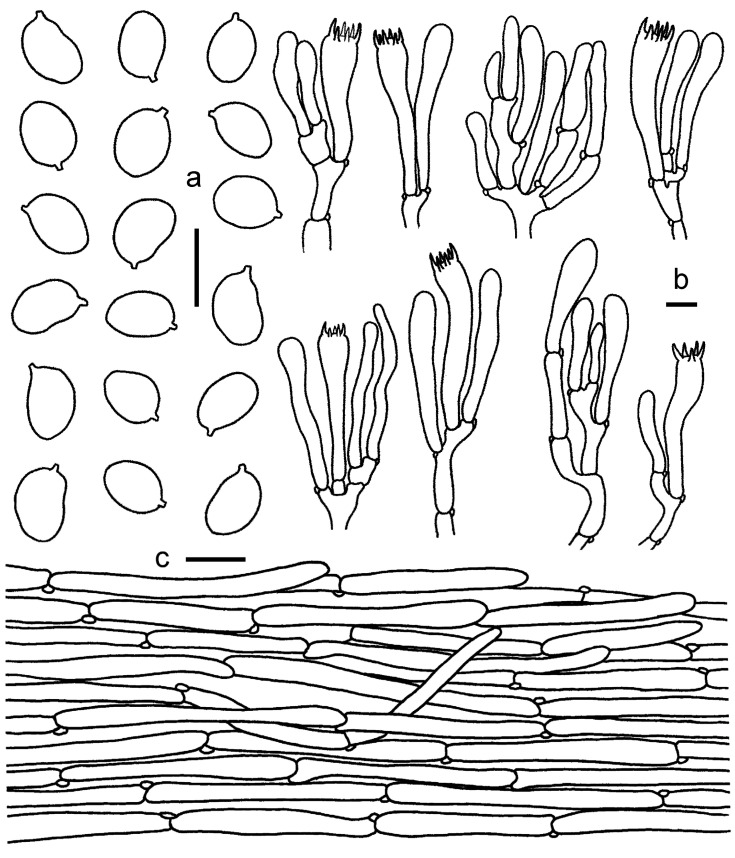
*Cantharellus sinocinnabarinus*. (**a**) Basidiospores. (**b**) Basidia, basidiola and elements of the subhymenium. (**c**) Pileipellis. Bars: (**a**,**b**) = 10 μm; (**c**) = 20 μm.

***Cantharellus sinocinnabarinus*** Ming Zhang, S.C. Shao & T.H. Li sp. nov.; Figure 4 and Figure 5.

MycoBank: MB843658.

GenBank: OM978984 for LSU, ON119099 for *tef1* and ON119048 for *rpb2*.

Etymology—Refers to the species distributed in China and is similar to *C. cinnabarinus* in morphology.

Diagnosis—This species is characterized by its small basidiomata, reddish orange to yellowish red pileus covered with white minute fibrils, yellowish orange to orange hymenophore and elongate elliptical basidiospores measuring (6.5–) 7–8 (9) × (4.5) 5–6 μm.

Type—China. Yunnan Province, Lijiang City, Yulong County, Jiuhe Village, 1 September 2020, alt. 2400 m, Ming Zhang (GDGM83230).

Basidiomata small-sized. Pileus 5–15 mm broad, applanate with a depressed center, not perforate; margin slightly incurved when young, applanate to reflexed with age; surface dry, orange, reddish orange to yellowish red (6A7–8A7), locally with white minute fibrils. Context thin, 0.5–1.5 mm thick, fleshy to fibrous, yellowish orange to reddish orange, unchanging when bruised. Hymenophore subdecurrent, with a clearly delimitation from stipe surface; lamellate ridges subdistant to close, well-developed, 1–2 mm high, appropriately bifurcate, with low interconnected low venose folds, particularly at pileus margin, yellowish orange to orange (4A7–6A7), unchanging when bruised. Stipe 10–15 mm long, 1–2.5 mm thick, subcylindrical, slightly tapering downward, glabrous or with obscure white minute fibrils, hollow, concolorous with pileus. Odor pleasant.

Basidiospores (100/4/4) (6.5)7–8(9) × (4.5)5–6 μm, L_m_ × W_m_ = 7.47(±0.5) × 5.21(±0.39) μm, Q = (1.25)1.27–1.6(1.67), Q_m_ = 1.43 ± 0.09; elliptical to elongate elliptical. Basidia 50–75 × 10–12 μm, clavate, with 4–8 sterigmata. Pileipellis a cutis, composed of procumbent hyphae; hyphae 4–13 μm in diam., colorless, thin-walled. Hymenophoral trama composed of cylindrical hyphae 5–10 μm in diam. Stipitipellis a cutis, composed of procumbent, branched hyphae; hyphae 4–12 μm in diam., mostly 7 μm in diam. Cystidia absent. Clamp connections common.

Habitat and distribution—Gregarious on soil in subalpine mixed forest dominated by *Cyclobalanopsis delavayi* (Franch.) Schott. and *Pinus yunnanensis* Franch. Currently known from southwest China.

Additional specimens examined—China. Yunnan Province, Jianchuan County, Qianshi Mountain, 7 September 2009, alt. 2491 m, Yu23 (HKAS58243); Lijiang City, Yulong County, Jiuhe Village, 1 September 2020, alt. 2400 m, Ming Zhang (GDGM83229, GDGM83232, GDGM83027), Li-Qiang Wu (GDGM83238).

Notes—*Cantharellus* *sinocinnabarinus* can be easily recognized in the field by its small reddish orange basidiomata. Morphologically, *C. sinocinnabarinus* is similar to *C. cinnabarinus*, *C. persicinus* R.H. Petersen and *C. texensis*. However, the latter three species were all originally reported from North America; *C. cinnabarinus* and *C. persicinus* differ in their larger basidiomata (pileus up to 40 mm), thicker-walled hyphae of pileipellis terminal cells, and different sizes of basidiospores (6.7–7.57 × 3.82–4.68 μm for *C. cinnabarinus*, and 10.2–11.9 × 6.3–7.2 μm for *C. persicinus*) [32]; *C. texensis* differs in its robust basidiomata and longer but narrower basidiospores (8–8.95 × 3.7–4.3 μm), with a larger Q value (1.8–2.2) [32].

Shao et al. [20] has described a specimen (HKAS58243) under the name *C. cinnabarinus* on the basis of the LSU sequence, which is geographically close to *C. sinocinnabarinus* in southwest China. In this study, the specimen (HKAS58243) was re-examined; the morphological features and molecular phylogenetic analyses all demonstrated that it is actually *C. sinocinnabarinus*.

In the multi-locus phylogentic trees, specimens of *C. sinocinnabarinus* formed a well-supported independent terminal branch (BS = 100%, BPP = 1.0) in the subg. *Cinnabarinus*, and are closely related to *C. cinnabarinus*. However, they can be easily distinguished by the morphological features and large genetic distance.

**Figure 6:**
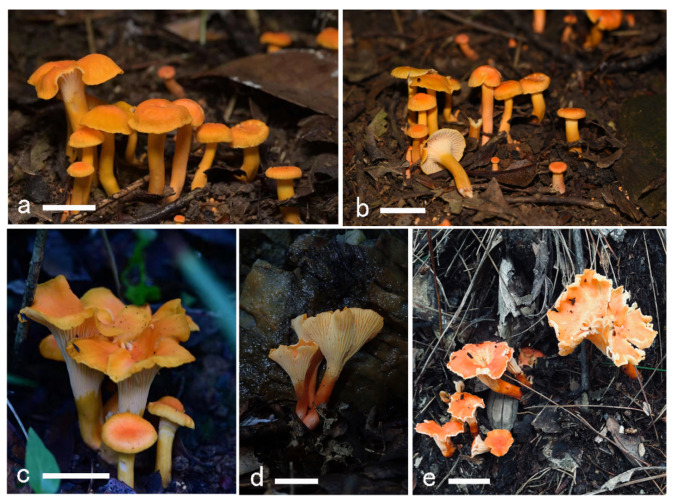
Basidiomata of *Cantharellus albovenosus*. (**a**,**b**) GDGM85852. (**c**,**d**) GDGM85846. (**e**) GDGM85142. Bars = 2 cm.

**Figure 7:**
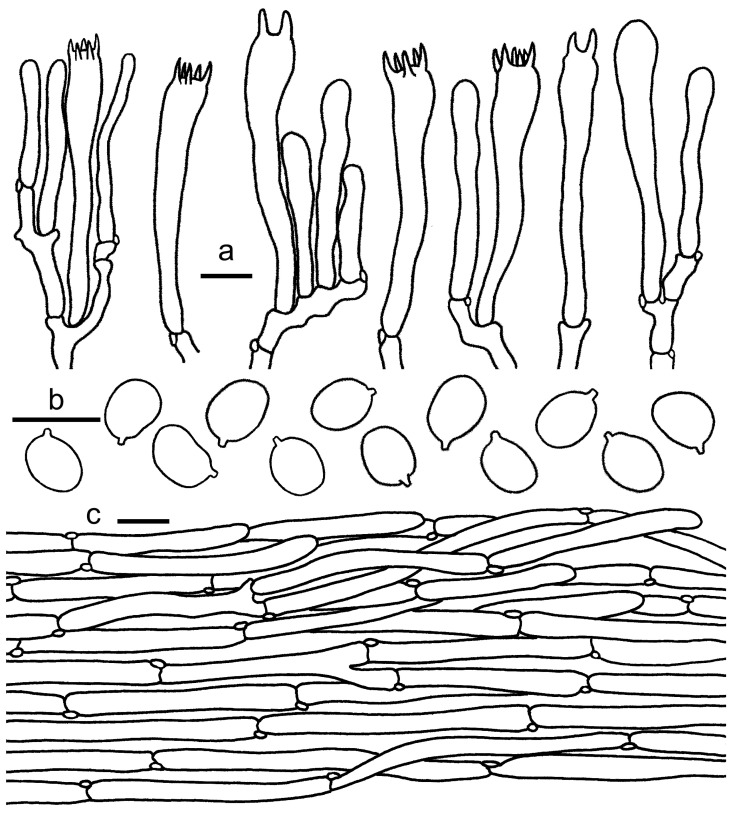
*Cantharellus albovenosus*. (**a**) Basidia, basidiola and elements of the subhymenium. (**b**) Basidiospores. (**c**) Pileipellis. Bars: (**a**,**b**) = 10 μm; (**c**) = 20 μm.

***Cantharellus albovenosus*** Buyck, Antonín & Ryoo, in Antonín, Hofstetter, Ryoo, Ka and Buyck, Mycol. Progr. 16(8): 757 (2017); Figure 6 and Figure 7.

Basidiomata small-sized. Pileus 20–55 mm broad, convex at first, then broad applanate with a depressed centre, subinfundibuliform when mature or old; margin inflexed to straight when young, then undulate; surface tomentose when young, then glabrescent and radially (innately) fibrillose to finely striate and rugulose, orange, deep orange to reddish orange (5A6–7A6, 5A8–7A8), then pallescent to light orange at margin. Hymenophore decurrent, with a clearly delimitation from stipe surface; lamellate ridges, subdistant to distant, relatively well-developed, 1–1.5 mm high, appropriately bifurcate and interconnected with low veined folds, particularly towards pileus margin, white to orange white (5A2–6A2), unchanging when bruised. Stipe 25–50 × 2.5–9 mm, cylindrical and slightly clavate to bulbose at base, finely tomentose when young, then glabrous or with finely longitudinally fibrillose, concolorous with pileus, orange to reddish orange, sometimes paler to light orange in some specimens. Context white, orangish under pileipellis, solid, becoming hollow-fibrous in stipe. Odor spicy. Taste mild.

Basidiospores 7–8.5 × 5–6 μm, L_m_ × W_m_ = 7.9(±0.48) × 5.5(±0.34) μm, Q = (1.33)1.4–1.5(1.54), Q_m_ = 1.44 ± 0.05; ellipsoid to subglobose, thin-walled, sometimes with granulose contents. Basidia 48–63 × 7–9 μm, 2–6-spored, clavate, sometimes subcapitate. Hymenial trama hyphae cylindrical to subinflated, sometimes irregular, thin-walled, 3–8 μm wide. Pileipellis a cutis composed of cylindrical, rarely subinflated, thin-walled, 4–10 μm wide hyphae; terminal cells 37–87 × 5–8 μm, adpressed, cylindrical, clavate or subfusoid. Stipitipellis a cutis of cylindrical, parallel, thin-walled, clamped, 3–7 μm wide hyphae.

Habitat and distribution—Scattered or gregarious on soil under mixed forest dominated by Fagaceae trees. Known to be from eastern China and Korea.

Specimens examined—China. Jiangsu Province, Nanjing City, Purple Mountain, 19 June 2021, alt. 150 m, Zi-Hang Zhang (GDGM85846); same location, 28 June 2021, Zi-Hang Zhang (GDGM85852, GDGM85853); Anhui Province, Huangshan National Scenic Area, 26 August 2021, alt. 1400 m, Chen-Jie Jiang (GDGM85142). Zhejiang Province, Lishui City, Jingning Town, Wangdongyang Alpine Wetland Nature Reserve 22 September 2016, Rui-Lin Zhao (HMAS279296, HMAS279292); same location, 23 September 2016, Rui-Lin Zhao (HMAS279262, HMAS279284).

Notes—*Cantharellus albovenosus*, recently reported from South Korea, is characterized by the combined features of the orange to reddish orange pileus, white to orange white and relatively well-developed lamellate hymenophore, the orange to reddish orange stipe, and the ellipsoid to nearly globose basidiospores (7–8.5 × 5–6 μm) [11]. Phylogenetically, *C. albovenosus* and *C. phloginus* clustered together in an almost similar phylogenetic position, and cannot be separated in our multi-locus phylogenetic tree (Figure 1). Morphologically, *C. phloginus* can be distinguished by its pastel red to pastel pink pileus and stipe, pale yellow to yellowish orange hymenophore and large basidiospores [6.8–9.5 (–12) × 5–7 μm] [22]. Ecologically, *C. albovenosus* is known from subtropical regions of South Korea and eastern China*;* meanwhile, *C. phloginus* is currently only known from tropical regions of southwest China. The distinguishable morphological features and different growth habits supported them as two distinct species, but some more effective molecular markers are needed to distinguish the two species.

**Figure 8:**
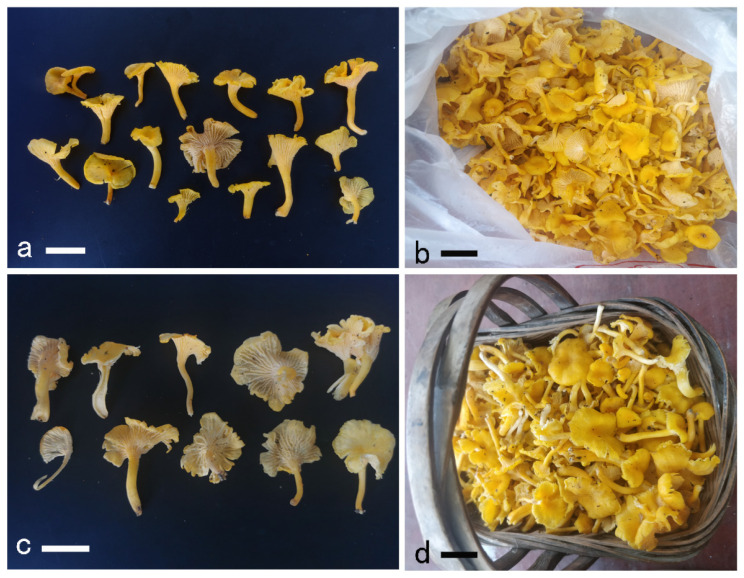
Basidiomata of *Cantharellus citrinus*. (**a**,**b**) GDGM86143. (**c**) GDGM86141. (**d**) GDGM80723. Bars = 2 cm.

**Figure 9:**
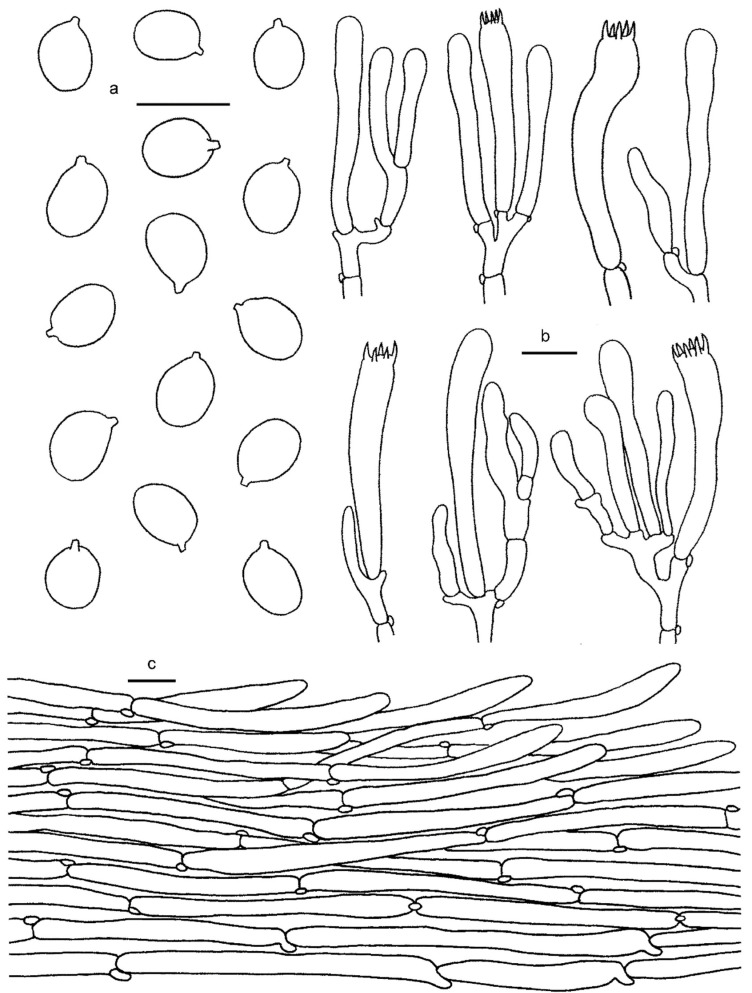
*Cantharellus citrinus*. (**a**) Basidiospores. (**b**) Basidia, basidiola and elements of the subhymenium. (**c**) Pileipellis. Bars: (**a**,**b**) = 10 μm; (**c**) = 20 μm.

***Cantharellus citrinus*** Buyck, R. Ryoo & Antonín, in Buyck, Hofstetter, Ryoo, Ka and Antonín, MycoKeys 76: 35 (2020); Figure 8 and Figure 9.

Basidiomata small-sized. Pileus 15–45 mm broad, convex, with involute margin when young, then gradually to broadly infundibuliform with depressed center, irregularly undulate or slightly cracked margin when old; surface dry or hygrophanous, glabrous or finely subtomentose, greenish yellow, light yellow, yellow to yellowish orange (1A4–4A4, 1A7–4A7). Context yellowish white, 1 mm thick in the center of the pileus, sharply attenuate towards margin, unchanging when exposed. Hymenophore decurrent, subdistant, composed of bifurcate, less than 1 mm high veined folds, particularly towards pileus margin, white to yellowish white (1A2–3A2), unchanging when bruised. Stipe 15–30 × 3–5 mm, central, cylindrical or slightly tapering towards base, hollow, glabrous, concolorous with pileus or paler, unchanging when handled. Odor fruity and pleasant. Taste mild.

Basidiospores 7–9 × 5–6(6.5) μm, L_m_ × W_m_ = 7.77(±0.47) × 5.29(±0.40) μm, Q = (1.17)1.23–1.6(1.64), Q_m_ = 1.47 ± 0.11; broadly elliptical to subglobose, smooth, guttulate, thin-walled. Basidia 55–65 × 7–8 μm, 4–6-spored, narrowly clavate, colorless to hyaline in KOH; sterigmata 5–10 μm long. Pileipellis a cutis with long, repent and occasionally interwoven hyphae, subcylindrical cells that are 5–15 μm wide, thin-walled. Stipitipellis a cutis of cylindrical, parallel hyphae, 5–10 μm wide. Clamp connections abundant in all tissues.

Habitat and distribution—Gregarious on soil under mixed forests in southwest China. Known from southwest China and Korea.

Specimen examined—China. Guizhou Province, Guiyang City, Longli County, Guanyin Village, bought from a wild mushroom market, 1 July 2020, alt. 1000 m, Ming Zhang (GDGM80825); Same location, 16 June 2020, Ting Li (GDGM80724, GDGM80723); 7 July 2021, Ming Zhang (GDGM86140, GDGM86141, GDGM86142, GDGM86143).

Notes—*Cantharellus citrinus*, recently reported from Korea [11], is characterized by its small basidiomata, greenish yellow to yellowish orange pileus, white to yellowish white hymenophore strongly bifurcate at pileus margin, glabrous and hollow stipe, and broadly elliptical to subglobose basidiospores [7–9 × 5–6 (6.5) μm]. In the multi-locus phylogentic tree, samples of *C. citrinus* formed a well-supported monophyletic terminal clade, and can be easily distinguished from other *Cantharellus* species.

Morphologically, *C. citrinus* might be easily identified as a species in the subg. *Parvocantharellus* by the small basidioma with a greenish yellow to yellowish orange pileus, and similar to *C. galbanus* Ming Zhang, C.Q. Wang & T.H. Li and *C. luteovirens* Ming Zhang, C.Q. Wang & T.H. Li. However, *C. galbanus*, recently reported from tropical China, differs in its smaller basidiomata, relatively well-developed hymenophore, and smaller basidiospores (6–7.5 × 4.8–5.5 µm) [13]; *C. luteovirens,* recently reported from subtropical China, differs in its yellow to yellowish-orange pileus, yellowish white to pale yellow hymenophore and smaller basidiospores (6–7.5 × 4.5–6 µm) [13].

**Figure 10:**
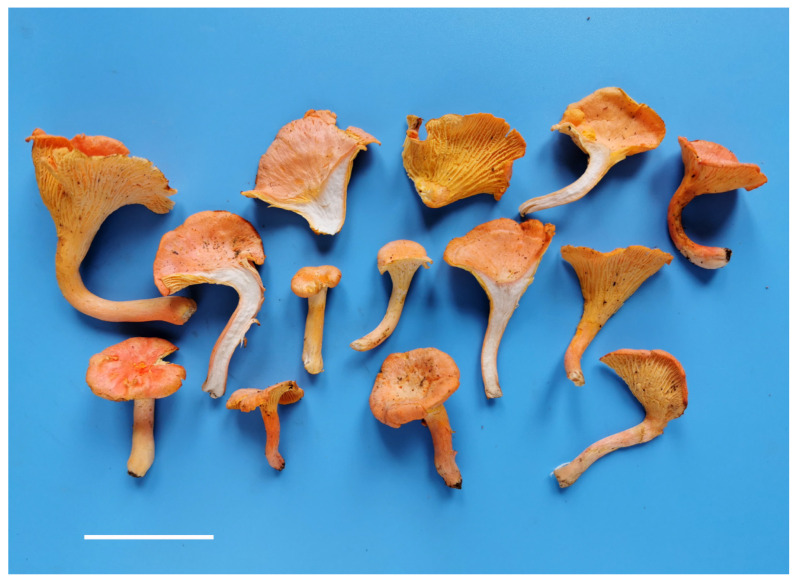
Basidiomata of *Cantharellus phloginus* (GDGM79007). Bar = 5 cm.

**Figure 11:**
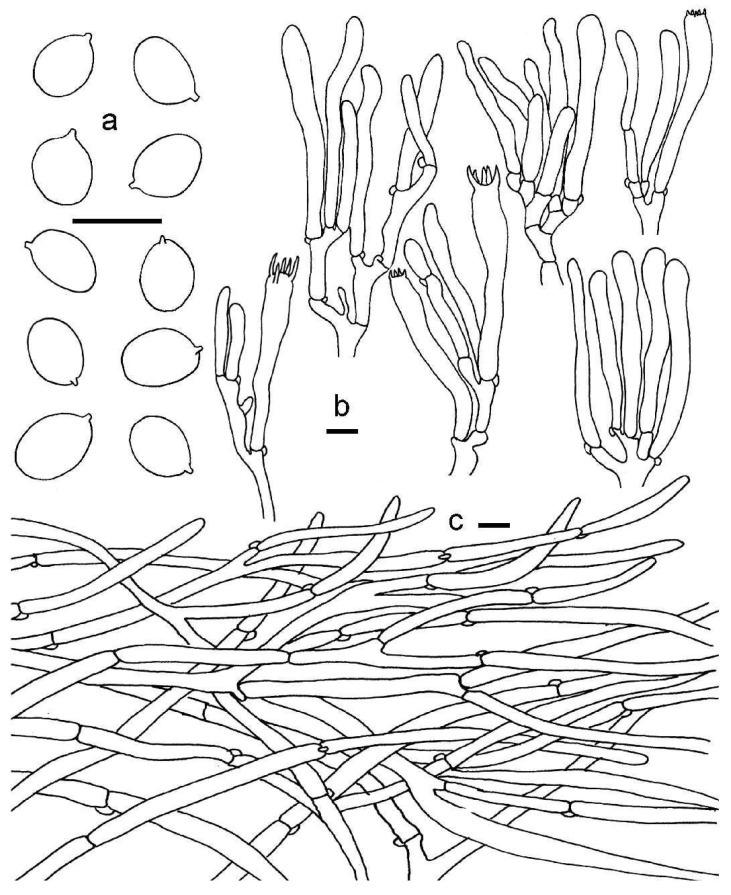
*Cantharellus phloginus*. (**a**) Basidiospores. (**b**) Basidia, basidiola and elements of the subhymenium. (**c**) Pileipellis. Bars: (**a**,**b**) = 10 μm; (**c**) = 20 μm.

***Cantharellus phloginus*** S.C. Shao & P.G. Liu, in Shao, Buyck, Tian, Liu and Geng, Mycoscience 57(2): 146 (2016); Figure 10 and Figure 11.

Basidiomata small to medium-sized. Pileus 20–60 mm broad, applanate with a concave center, margin incurved at first, then becoming applanate or slightly reflexed with age, glabrous, pastel red to pastel pink (7A4–11A4); Context 2–3 mm thick, white, with pinkish hues under pileipellis, unchanging when bruised; Hymenophore decurrent, well-developed, lamellate ridges with anastomosing veins, forking towards pileus margin, pale yellow to light yellow (3A3–4A3), unchanging when touched. Stipe 20–40 × 4–8 mm, central, solid, subcylindrical, or slightly tapering towards base, glabrous, concolorous with pileus or paler to pinkish with yellowish hues, unchanging when handled. Odor fruity. Taste pleasant.

Basidiospores 6.8–9.5 (–12) × 5–7 μm, L_m_ × W_m_ = 8.49(±1.09) × 5.71(±0.69) μm, Q = (1.33)1.36–1.6(1.7), Q_m_ = 1.49 ± 0.18; broadly ellipsoid to subglobose, smooth, guttulate. Basidia 60–95 × 8–10 μm, 2–6-spored, narrowly clavate, colorless to hyaline in KOH; sterigmata 3–7 μm long. Hymenophoral trama composed of cylindrical interwoven hyphae 3–13 μm in diam. Pileipellis a subcutis, composed of long, repent, branched, and slightly interwoven hyphae, with subcylindrical cells in 3–13 μm wide, thin-walled. Clamp connections abundant in all tissues.

Habitat and distribution—Gregarious or caespitose under mixed forests, dominated by *Pinus* sp. and *Castanopsis* in the tropical forest. Currently known to be southwest China.

Specimens examined—China. Yunnan Province, Puer City, alt. 1500 m, 26 August 2009, S.C. Shao 98 (HKAS58208, holotype); Puer City, bought from a mushroom market, alt. 1500 m, 28 September 2019, Ming Zhang (GDGM79007).

Notes—*Cantharellus phloginus*, recently reported from southwest China, is characterized by its pastel red to pastel pink pileus and stipe, pale yellow to yellowish orange, well-developed hymenophore, and ellipsoid basidiospores [6.8–9.5 (–12) × 5–7 μm] [22]. Morphologically, *C. phloginus* is similar to *C. cinnabarinus* and *C. texensis* Buyck & V. Hofst with the pinkish red pileus color. However, *C. cinnabarinus* differs in its small basidiomata, reddish pink pileus, small basidiospores [(6.4) 6.7–7.5 (8.1) × (3.7) 3.8–4.6 (5.2) μm] and thick-walled pileipellis [32]; *C. texensis* differs in its slender basidiomata, reddish pink pileus, relatively well developed hymenophore, small basidiospores [8–8.95 (9.4) × (3.3) 3.7–4.3 μm], and thinner-walled pileipellis that is faintly covered with zebroid incrustation [32]. Ecologically, *C. phloginus* occurs under trees of *Pinus* sp. and *Castanopsis* sp. in tropical regions of southwest China, while *C. cinnabarinus* and *C. texensis* occur on sandy loam in oak-pine forests in temperate regions of North America [32].

#### 3.2.2. *Cantharellus* subgen. *Parvocantharellus* Eyssart. & Buyck

**Figure 12:**
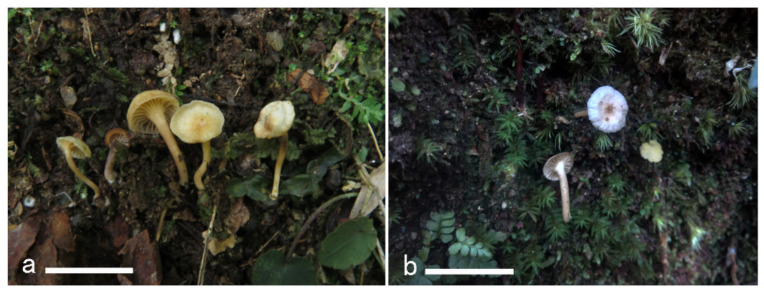
Basidiomata of *Cantharellus convexus*. (**a**) GDGM70307. (**b**) GDGM54841. Bars = 2 cm.

**Figure 13:**
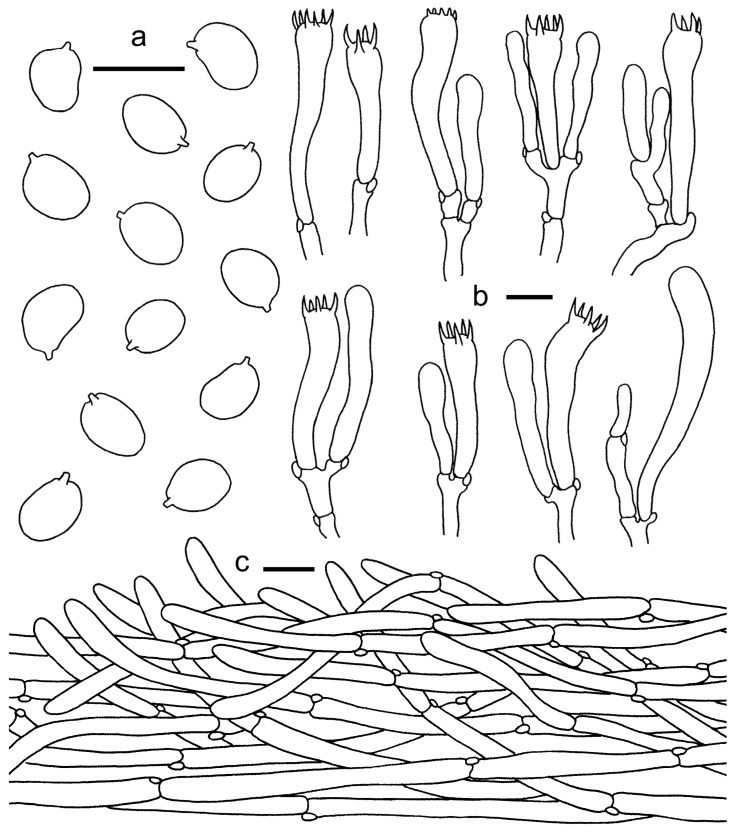
*Cantharellus convexus*. (**a**) Basidiospores. (**b**) Basidia, basidiola and elements of the subhymenium. (**c**) Pileipellis. Bars: (**a**,**b**) = 10 μm; (**c**) = 20 μm.

***Cantharellus convexus*** Ming Zhang & T.H. Li sp. nov.; Figure 12 and Figure 13.

MycoBank: MB843659.

GenBank: OM978941 for LSU, ON119053 for *tef1* and ON119037 for *rpb2.*

Etymology— “*convexus*” refers to the convex of the pileus center.

Diagnosis—This species can be easily distinguished from others in *Cantharellus* by its small basidiomata, yellowish white pileus, distant and well-developed lamellate hymenophore with or without bifurcate low veins and smaller basidiospores at 6–7 × 4.5–5 μm.

Type—China. Guangdong Province, Shaoguan City, Nanling National Nature Reserve, alt. 800 m, 29 July 2017, Ming Zhang (GDGM70307).

Basidiomata small-sized. Pileus 5–12 mm broad, convex when young, then gradually to nearly applanate with a central shallow depression at maturity; surface dry, tomentosus, mostly yellowish white, pale yellow to pale orange (2A2, 2A3–5A3), but in some specimens can be yellowish brown to brown, with a deeper center to olive brown to yellowish brown (4E5–5E5); margin wavy, incurved when young, decurved to slightly upturned at maturity, unchanging when handled. Context yellowish white, thin, unchanging when exposed. Hymenophore decurrent, lamellate ridges distant, relatively well developed, occasionally forking towards pileus margin, with or without bifurcate low veins between ridges, yellowish white to pale yellow (2A2–4A2, 2A3–4A3), unchanging when bruised. Stipe 10–20 × 1.5–3 mm, central, cylindrical or slightly tapering towards base, glabrous or faintly scaly, concolorous with pileus or paler, unchanging when handled. Odor not distinct.

Basidiospores (50/2/2) 6.0–7.0 × 4.5–5.0 μm, L_m_ × W_m_ = 5.71(±0.64) × 4.87(±0.49) μm, Q = (1)1.1–1.27(1.37), Q_m_ = 1.17 ± 0.07, broadly ellipsoid to subglobose, smooth, guttulate. Basidia 32–50 × 7–9 μm, 4–6-spored, narrowly clavate, colorless to hyaline in KOH, sterigmata 3–7 μm long. Hymenophoral trama irregular, composed of colorless and branched hyphae, 5–22 μm wide, septate, thin-walled. Pileipellis a cutis with long, repent, branched, and usually interwoven hyphae consisting of subcylindrical cells in 3–15 μm wide, thin-walled; terminal cells appressed to suberect, mostly cylindrical, up to 110 μm long, 5–15 μm wide. Stipitipellis a cutis of cylindrical, parallel hyphae, 3–10 μm wide; terminal cells clavate or cylindrical. Clamp connections abundant in all tissues.

Habitat and distribution—Gregarious or scattered under broadleaf forests (dominated by Fagaceae trees) in subtropical China. Currently known from Guangdong and Hunan Province, Southern China.

Additional specimens examined—China. Hunan Province, Chenzhou City, Sanjiangkou Town, Jiulongjiang National Forest Park, under *Castanopsis hystrix* mixed with other broadleaf trees, alt. 200 m, 3 August 2017, Ming Zhang (GDGM54841).

Notes—*Cantharellus convexus* is characterized by its small basidiomata, convex pileus covered with fibrillose scales, distant and well-defined lamellate hymenophore without anastomosis between the folds, broad elliptic to subglobose basidiospores and thin-walled hyphae of the pileipellis. These traits taxonomically enable the placement of *C. convexus* into subg. *Parvocantharellus*.

Phylogenetically, two specimens of *C. convexus* formed an isolated lineage in subg. *Parvocantharellus*, and are closely related to *C. tabernensis*. A BLAST result of ITS sequence in the GenBank database also demonstrated that the similarity between *C. convexus* and *C. tabernensis* (JN944012, O7.064) is 93.7%. However, *C. tabernensis,* originally reported from North America, differs in its more robust basidiomata, dull orange-yellow to yellowish-brown pileus, vivid orange-yellow hymenophore and stipe and larger basidiospores (6–9 × 4.4–5.9 µm) [40]. Additionally, *C. tabernensis,* currently only known from Texas, Louisiana and Mississippi in North America, occurs in well-drained (sandy) soil in mixed woods, and near to *Pinus elliottii* Engelm. Meanwhile, *C. convexus* was found in broadleaf forests in southern China, close to Fagaceae trees. Another North America species, *C. appalachiensis,* also demonstrates a close relationship with *C. convexus*. However, *C. appalachiensis* differs in its larger and more robust basidiomata, with a drab yellow to dull brown pileus applanate with the center depressed, surface locally dull-grayish due to aggregate minute fibrils and with larger basidiospores (6.6–8.9 × 4.4–5.9 µm) [41,42].

Morphologically, *C. convexus* is similar to *C. austrosinensis* Ming Zhang, C.Q. Wang & T.H. Li, *C. koreanus* Buyck, Antonín & Ryoo and *C. luteovirens*. However, *C. austrosinensis* differs in its pastel yellow to greyish-yellow pileus, usually with a greyish-orange to brownish-orange center, broader basidiospores (6–8 × 4.8–6 µm) and strictly associated with coniferous trees (*Pinus massoniana*) [13]; *C. koreanus*, originally described from the temperate region of the Republic of Korea, differs in its dirty yellow-brown to pale brown pileus usually with a brown to dark brown center and larger basidiospores [6–8 (–9) × 4.2–5.5 (–6.5) μm] [11]; *C. luteovirens* differs in its yellow to orange pileus, greyish-yellow to greyish-orange hymenophores, broadly ellipsoid to subglobose basidiospores (7–8 × 5.2–6.5 μm) and is currently only found be associated with *Acacia* trees [13].

**Figure 14:**
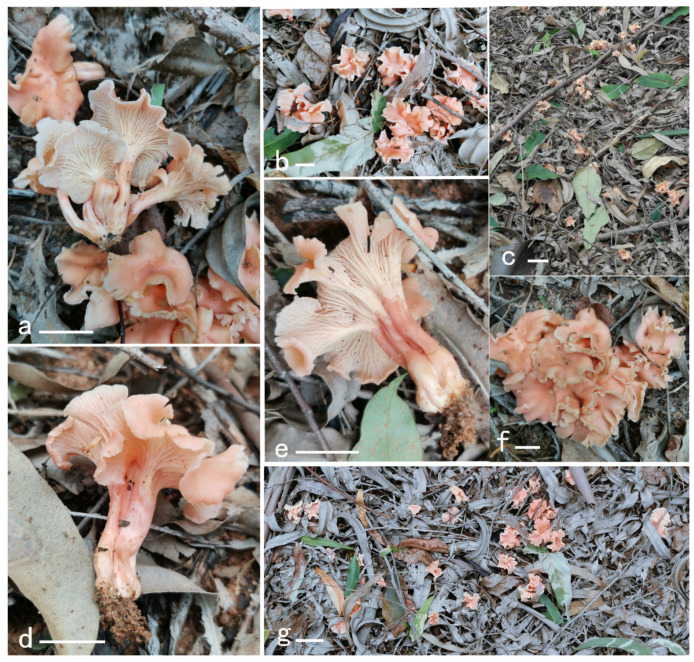
Basidiomata of *Cantharellus neopersicinus*. (**a**–**e**) GDGM87366. (**f**,**g**) GDGM85145. Bars: (**a**,**b**,**d**–**f**) = 2 cm; (**c**,**g**) = 5 cm.

**Figure 15:**
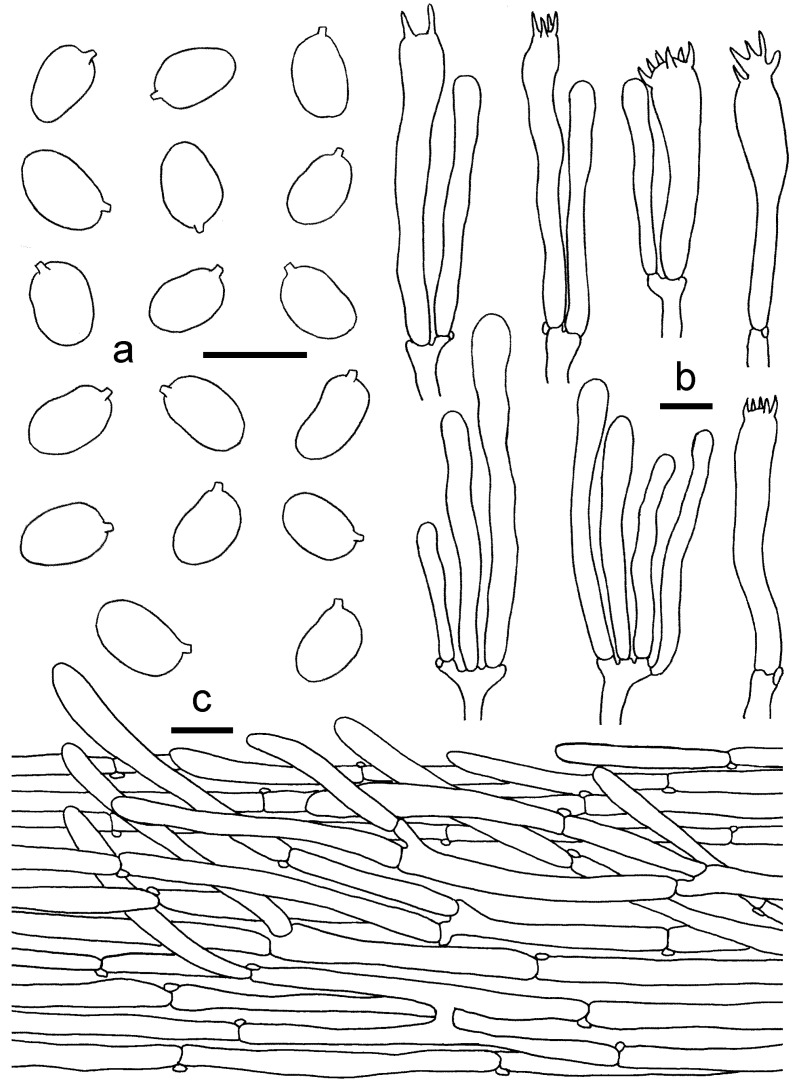
*Cantharellusneopersicinus*. (**a**) Basidiospores. (**b**) Basidia, basidiola and elements of the subhymenium. (**c**) Pileipellis. Bars: (**a**,**b**) = 10 μm; (**c**) = 20 μm.

***Cantharellus neopersicinus*** Ming Zhang, T.H. Li & X.Y. Chen sp. nov. Figure 14 and Figure 15.

MycoBank: MB843660

GenBank: OM978943 for LSU, ON119057 for *tef1* and ON119042 for *rpb2*

Etymology—refers to the color similar to *Cantharellus persicinus*.

Diagnosis—The pastel red to pink pileus, white to pinkish hymenophore with strongly bifurcate low veins and ellipsoid to subglobose [(6–)7–8.5(–9) × (4–)4.5–5.5(–6) μm], make *C. neopersicinus* easily distinguished from other species in the subg. *Parvocantharellus*.

Type—China. Guangdong Province, Leizhou City, Fangcha Village, under Eucalyptus robusta, alt. 105 m, 16 October 2021, Xiu-Yuan Chen (GDGM87366).

Basidiomata small-sized. Pileus 15–45 mm broad, convex when young, then gradually to nearly applanate with a central shallow depression at maturity; surface dry, glabrous, pastel red, pastel pink to pink (8A4–12A4); margin incurved when young, reflexed with age, wavy, sometimes irregularly split; unchanging when touched. Context thin, reddish white or pinkish (8A2–12A2), unchanging when exposed. Hymenophore decurrent, but clearly demarcated with stipe, lamellate ridges close to subdistant, poorly-developed, strongly forking towards pileus margin, with bifurcate low veins between ridges, white to pinkish, unchanging when bruised. Stipe 15–40 × 3–8 mm, central, cylindrical or slightly tapering towards base, hollow, glabrous, concolorous with pileus, unchanging when handled. Odor fruity. Taste mild.

Basidiospores (50/2/2) (6–)7–8.5(–9) × (4–)4.5–5.5(–6) μm, L_m_ × W_m_ = 7.78(±0.64) × 4.871(±0.46) μm, Q = (1.2)1.4–1.77(2), Q_m_ = 1.6 ± 0.15, ellipsoid to subglobose, smooth, guttulate. Basidia 45–62 × 7–9 μm, 4–6-spored, narrowly clavate, colorless to hyaline in KOH, sterigmata 3–7 μm long. Hymenophoral trama irregular to subregular, composed of colorless and branched hyphae, 8–16 μm wide, septate, thin-walled. Pileipellis a cutis with long, repent to suberect, branched, and slightly interwoven hyphae, subcylindrical cells in 8–15 μm wide, thin-walled; terminal cells appressed, mostly cylindrical, up to 100 μm long, 5–15 μm wide. Stipitipellis a cutis of cylindrical, parallel hyphae, 3–8 μm wide, terminal cells cylindrical. Clamp connections abundant in all tissues.

Habitat and distribution—Gregarious or scattered under *Eucalyptus robusta* Smith in tropical China. Currently known from Guangdong Province, Southern China.

Additional specimens examined—China. Guangdong Province, Leizhou City, Fangcha Village, alt. 105 m, 25 October 2021, Xiu-Yuan Chen (GDGM85145).

Notes—*Cantharellus neopersicinus* is characterized by its small basidiomata, pastel red to pink pileus, poorly-developed lamellate hymenophore with strongly bifurcate low veins and ellipsoid to subglobose basidiospores [(6–) 7–8.5 (–9) × (4–) 4.5–5.5 (–6) μm]. Phylogenetic analyses based on multi-locus datasets demonstrated that *C. neopersicinus* was well nested into the subg. *Parvocantharellus*, formed a well-supported terminal clade, and was closely related to *C. albus* S.P. Jian & B. Feng and *C. luteolus*. However, *C. albus*, recently reported from China, can be easily distinguished by its white basidiomata slightly changing to yellowish when bruised, a spicy taste and smaller basidiospores (5.5–7.5 × 4.5–6 µm) [12,13]; *C. luteolus* differs in its small basidiomata, yellow to orange pileus, greyish-yellow to greyish-orange hymenophore and oval to subglobose basidiospores (7–8 × 5.2–6.5 μm) [13].

Morphologically, the pastel red to pink pileus color is easily reminiscent of the species *C. cinnabarinus*, *C. coccolobae* Buyck, P.-A. Moreau & Courtec., *C. phloginus* and *C. persicinus*. However, the former three species belong to the subg. *Cinnabarinus*, and can be easily distinguished from *C. neopersicinus* by the genetic distances. Besides, *C. cinnabarinus* differs in its cinnabar red to bright orange pileus, thick-walled hyphal terminal cells of pileipellis and smaller basidiospores (6.7–7.57 × 3.82–4.68 μm) [32]. *Cantharellus coccolobae* differs in its salmon orange hymenophore, white stipe context partly changing to yellowish when cut, large basidiospores [(7.9) 8.3–9.3 (9.8) × (4.8) 5.3–5.9 (6) µm], longer basidia up to 120 µm and the thick-walled hyphae of the pileipellis. Additionally, *C. coccolobae* was reported to be strictly associated with *Coccoloba* trees, while *C. neopersicinus* is under *Eucalyptus* trees [33]. *Cantharellus phloginus* is redescribed in this study and differs in its darker pileus color, pale yellow to light yellow hymenophore, white context and larger basidiospores [6.8–9.5 (–12) × 5–7 μm]. *Cantharellus persicinus,* originally reported from North America, differs in its more robust basidiomata, larger basidiospores (9.6–10.9 × 6.3–7.1 μm), and thick-walled cells of pileipellis. In addition, *C. persicinus* is reported to be associated with oaks or eastern hemlock [32,43,44].

**Figure 16:**
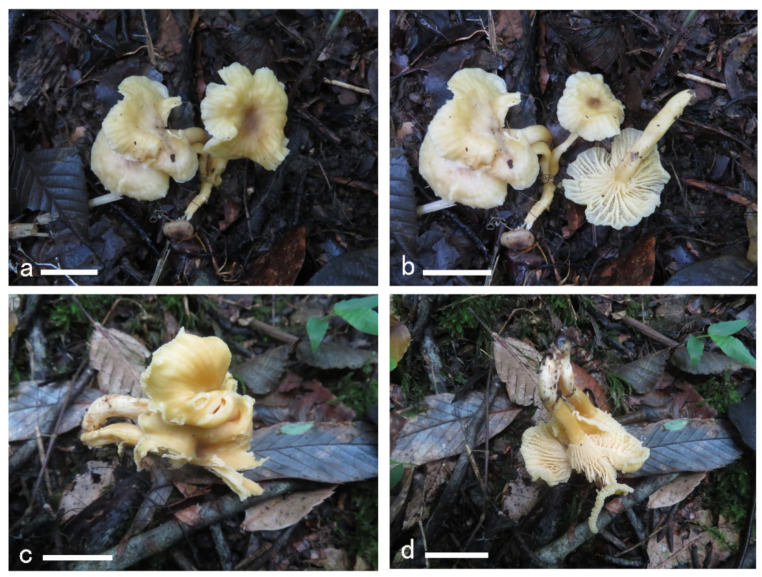
Basidiomata of *Cantharellus koreanus*. (**a**,**b**) GDGM79233. (**c**,**d**) GDGM85306. Bars = 2 cm.

**Figure 17:**
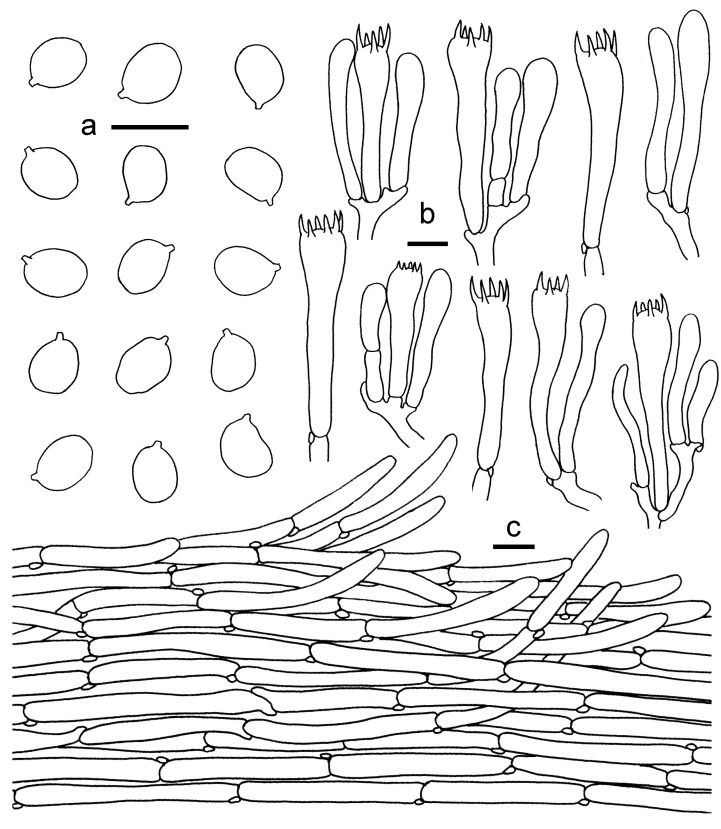
*Cantharellus koreanus*. (**a**) Basidiospores. (**b**) Basidia, basidiola and elements of the subhymenium. (**c**) Pileipellis. Bars: (**a**, **b**) = 10 μm; (**c**) = 20 μm.

***Cantharellus koreanus*** Buyck, Antonín & Ryoo, in Antonín, Hofstetter, Ryoo, Ka and Buyck, Mycol. Progr. 16(8): 755 (2017); Figure 16 and Figure 17.

Basidiomata small-sized. Pileus 15–40 mm broad, convex at first, then gradually applanate with slightly an umbilicate centre; margin involute at first, undulate; surface dry, glabrous or finely tomentose-fibrillose at centre, mostly pale yellow to light yellow (1A3–4A3,1A4–4A4), olive brown to light brown (4D4–5D4) at centre, with obscurely sulcate at margin. Hymenophore with lamellate ridges; ridges broadly adnate to subdecurrent, with a clearly delimitation from the stipe surface, well-developed, bifurcate and with interconnected low veins, up to 1 mm high, yellowish white (2A2–4A2), unchanging when bruised. Stipe10–40 mm long, 2–5 mm thick, subcylindrical to cylindrical, slightly enlarged downward, but sometimes tapering towards base, glabrous or with faintly scaly, hollow, concolorous with pileus, darker and more somber than lamellae ridges. Odor fruity. Taste mild.

Basidiospores 5–8 × (4–) 4.5–6 μm, L_m_ × W_m_ = 7.05(±0.51) × 5.192(±0.34) μm, Q = (1.08)1.2–1.45(1.6), Q_m_ = 1.36 ± 0.097, ellipsoid, broadly ellipsoid, thin-walled. Basidia 40–70 × 8–12 μm, 4–6-spored, narrowly clavate, sometimes subcapitate, thin-walled, clamped. Hymenophoral trama composed of clavate, subcylindrical, subregular, branched, thin-walled, clamped hyphae 5–12 μm wide. Pileipellis a cutis, composed of cylindrical, thin-walled hyphae, 5–15 μm wide; terminal cells clavate, fusoid to cylindrical, up to 100 μm long. Stipitipellis a cutis of cylindrical, parallel, branched, thin-walled hyphae 2–9 μm wide. Clamp connections abundant in all tissues.

Habitat and distribution—Gregarious or scattered under broadleaf forests (dominated by *Fagaceae* trees) in subtropical regions of China. Known from Hunan Province, China and Korea.

Specimens examined—China, Hunan Province, Zhangjiajie City, Zhangjiajie National Forest Park, alt. 1200 m, 17 July 2020, Wei-Qiang Qin (GDGM79233); same location, alt. 1100 m, 5 July 2021, Wei-Qiang Qin (GDGM85306).

Notes—*Cantharellus koreanus*, recently reported from Korea, is firstly reported from China in this study. It is characterized by the small basidiomata, the dirty yellow-brown to pale brown pileus with a brown to dark brown center, the well-development hymenophoral ridges with yellow tinge, and the ellipsoid to broadly ellipsoid basidiospores 6–8 (–9) × 4.2–5.5 (–6.5) μm in Antonín et al. [11] and 5–8 × (4–) 4.5–6 μm in this study.

Phylogenetically, *C. koreanus* is closely related to *C. appalachiensis*, *C. austrosinensis* and *C. tabernensis*. Indeed, *C. koreanus* is similar to *C. appalachiensis*, *C. austrosinensis* and *C. tabernensis* in morphology. However, *C. appalachiensis* differs in its larger and more robust basidiomata (pileus up to 50 mm broad), drab yellow to dull brown pileus, narrower basidia (5.5–9 μm in diam.), shorter and slightly thickened end cell of pileipellis, narrower hyphae of hymenophoral trama, and association with oaks and other hardwoods [41,45,46]; *C. austrosinensis* differs in its smaller basidiomata, pastel yellow to greyish-yellow pileus with a greyish-orange to brownish-orange center, shorter and narrower basidia (50–55 × 7–9 μm), interwoven hyphae of pileipellis, and symbiosis with coniferous trees [13]; *C. tabernensis* differs in its dull orange yellow to yellowish brown pileus, vivid orange yellow hymenophore and stipe, shorter and narrower basidia (35–55 × 5–8 μm), and distribution in North America [40,42,46].

In addition, several species were recently reported from China, and are also similar to *C. koreanus* in morphology, such as *C. galbanus*, *C. luteolus* Ming Zhang, C.Q. Wang & T.H. Li, *C. luteovirens* and *C. sinominor* Ming Zhang, C.Q. Wang & T.H. Li [13], but they can be easily separated from each other by the large genetic distances.

### 3.3. Key to Species of Subgenus Cinnabarinus in China

**1** Basidiomata with pastel red or reddish orange tinge...................................................2

**1’** Basidiomata without red tinge........................................................................................4

**2** Pileus: small, always <20 mm broad..............................................***C. sinocinnabarinus***

**2’** Pileus: relatively large, usually >20 mm wide...............................................................3

**3** Basidiospores: 7–8.5 × 5–6 μm..................................................................***C. albovenosus***

**3’** Basidiospores: 6.8–9.5 (–12) × 5–7 μm.........................................................***C.*** ***phloginus***

**4** Pileus: greenish yellow to yellowish orange, hymenophore white to yellowish white; basidiospores: 7–9 × 5–6(6.5) μm.....................................................................***C. citrinus***

**4’** Pileus: orange to orange-yellow, hymenophore pinkish white to orange white; basidiospores: 7.5–9 × 5–6.5 μm, basidia up to 100 μm....................................***C*. *chrysanthus***

## 4. Discussion

In this study, the species diversity of *C.* subg. *Cinnabarinus* from China were examined. Five species were identified based on morphological characters and multi-locus phylogenetic analyses, containing two new species *C. chrysanthus* and *C. sinocinnabarinus*, two newly recorded species *C. albovenosus* and *C. citrinus* to China, and a known species, *C. phloginus*. In addition, three species belonging to the subg. *Parvocantharellus* were firstly discovered from China, including two new species *C. convexus* and *C. neopersicinus*, and a new recorded species, *C. koreanus*.

In the past, the knowledge of species diversity of *Cantharellus* in China was poor and the specimens with large and yellow to orange basidiomata were mostly misidentified as the type species of the genus *C. cibarius*; meanwhile, specimens with small and yellow to orange red basidiomata were often inaccurately treated as *C. minor* Peck or *C. cinnabarinus*. However, a recent study proved that the distribution of *C. cibarius* is limited to northeast China, and the so-called “*C. cibarius*” reported from southwest China is actually *C. yunnanensis* W.F. Chiu [8]; meanwhile, the specimens labeled as “*C. minor*” in China were also proven to be misidentified, several new species with small basidiomata have been reported from China, and the distribution of *C. minor* with correctly identified specimens has not been found in China [13]. *Cantharellus cinnabarinus* was widely reported in China [19,21], but those photos of *C. cinnabarinus* used in the two literatures look like *C. albovenosus*; the correctly identified specimens of *C. cinnabarinus* in China have not been found in the present study. However, three morphologically similar species were discovered. The specimen HKAS58243 from southwest China, firstly identified as *C. cinnabarinus* in Shao et al. [20], was proven to be a native species of *C. sinocinnabarinus* in the present study. In addition, *C. sinocinnabarinus* seems to be restricted to subalpine habitats, and prefers symbiosis with *Cyclobalanopsis delavayi* and *Pinus yunnanensis*. The other two species, *C. albovenosus* and *C. phloginus,* are easily misidentified as *C. cinnabarinus* by their small basidiomata and reddish pileus color. However, *C. albovenosus*, recently reported from Korea, has been also found in eastern China, and *C. phloginus* seems to be restricted to tropical to subtropical regions in southwest China. Thus, we speculate that the specimens of “*C. cinnabarinus”* in Anhui, Guangdong, Jiangsu and Zhejiang provinces could be *C. albovenosus*, the distribution of “*C. cinnabarinus*” from tropical to subtropical regions of southwest China could be *C. phloginus* and the collections of “*C. cinnabarinus*” from subalpine regions of southwest China could be *C. sinocinnabarinus.*

*Cantharellus neopersicinus*, newly discovered in this study, is a remarkable species in *Cantharellus*. Morphologically, *C. neopersicinus* can be easily identified as a member of subg. *Cinnabarinus* or subg. *Cantharellus*, due to its pastel red to pink pileus and white to pinkish hymenophore; however, phylogenetic analyses demonstrated that it belongs to the subg. *Parvocantharellus*, which makes it the first species reported from China with pastel red to pink tinge in the subg. *Parvocantharellus*. Ecologically, *C. neopersicinus* is distributed in tropical areas of southern China, and currently, the only known symbiosis is with *Eucalyptus robusta*.

*Cantharellus* subg. *Parvocantharellus,* mainly composed of small-sized species, was suggested to be a monophyletic group, and closely related to the subg. *Cinnabarinus* [3]. However, in the present study, the subgenus was proven to be paraphyletic or polyphyletic; two species of *C. cyanoxanthus* R. Heim ex Heinem. and *C. subcyanoxanthus* Buyck, Randrianj. & Eyssart formed an isolated clade in the multi-locus phylogenetic tree, and could represent a separate generic clade. The result is similar to previous studies [13,16].

Species in the two subgenera are difficult to separate in morphology because most species share similar characteristics of small basidiomata, abundant clamps and thin-walled hyphal ends at the pileus surface. However, they formed two separate clades in the multi-locus phylogenetic trees, and can be easily distinguished by molecular phylogenetic evidence. In addition, the species in subg. *Cinnabarinus* mostly own distinct orange, pink or red tinge, and can be distinguished from subg. *Parvocantharellus*. In future work, more detailed morphological observations are needed to provide new evidences for distinguishing the two subgenera.

## Data Availability

Publicly available datasets were analyzed in this study. These data can be found here: (https://www.ncbi.nlm.nih.gov/; https://www.mycobank.org/page/Release%20names; accessed on 20 March 2022).

## References

[1] Fries E.M. (1821). Systema Mycologicum, Sistens Fungorum Ordines, Genera et Species.

[2] Kumari D., Upadhyay R.C., Reddy M.S. (2011). *Cantharellus pseudoformosus*, a new species associated with *Cedrus deodara* from India. Mycoscience.

[3] Buyck B., Kauff F., Eyssartier G., Couloux A., Hofstetter V. (2014). A multilocus phylogeny for worldwide *Cantharellus* (Cantharellales, Agaricomycetidae). Fungal Divers..

[4] Henkel T.W., Wilson A.W., Amie M.C., Dierks J., Uehling J.K., Roy M., Schimann H., Wartchow F., Mueller G.M. (2014). *Cantharellaceae* of Guyana II: New species of *Craterellus*, new South American distribution records for *Cantharellus guyanensis* and *Craterellus excelsus*, and a key to the neotropical taxa. Mycologia.

[5] De Kesel A.D., Amalfi M., Kasongo Wa Ngoy B., Yorou N.S., Raspé O., Degreef J., Buyck B. (2016). New and interesting *Cantharellus* from tropical Africa. Cryptog. Mycol..

[6] Ogawa W., Endo N., Fukuda M., Yamada A. (2018). Phylogenetic analyses of Japanese golden chanterelles and a new species description, *Cantharellus anzutake* sp. nov.. Mycoscience.

[7] Cao T., Hu Y.P., Yu J.R., Wei T.Z., Yuan H.S. (2021). A phylogenetic overview of the *Hydnaceae* (Cantharellales, Basidiomycota) with new taxa from China. Stud. Mycol..

[8] Shao S.C., Liu P.G., Wei T.Z., Herrera M. (2021). New insights into the taxonomy of the genus *Cantharellus* in China: Epityfication of *C. yunnanensis W.F. Chiu* and the first record of *C. cibarius* Fr. Cryptog. Mycol..

[9] Corner E.J.H. (1966). A Monograph of Cantharelloid Fungi.

[10] Buyck B., Kauff F., Cruaud C., Hofstetter V. (2013). Molecular evidence for novel *Cantharellus* (Cantharellales, Basidiomycota) from tropical African miombo woodland and a key to all tropical African chanterelles. Fungal Divers..

[11] Antonín V., Hofstetter V., Ryoo R., Ka K.H., Buyck B. (2017). New *Cantharellus* species from the Republic of Korea. Mycol. Prog..

[12] Jian S.P., Dai R., Gao J.U.N., Feng B. (2020). *Cantharellus albus*, a striking new species from Southwest China. Phytotaxa.

[13] Zhang M., Wang C.Q., Buyck B., Deng W.Q., Li T.H. (2021). Multigene Phylogeny and Morphology Reveal Unexpectedly High Number of New Species of *Cantharellus Subgenus Parvocantharellus* (Hydnaceae, Cantharellales) in China. J. Fungi.

[14] An D.Y., Liang Z.Q., Jiang S., Su M.S., Zeng N.K. (2017). *Cantharellus hainanensis*, a new species with a smooth hymenophore from tropical China. Mycoscience.

[15] Zhang Y.Z., Liang Z.Q., Xie H.J., Wu L.L., Xue R., Zeng N.K. (2021). *Cantharellus macrocarpus* (Cantharellaceae, Cantharellales), a new species from tropical China. Phytotaxa.

[16] Buyck B., Hofstetter V., Ryoo R., Ka K.-H., Antonín V. (2020). New *Cantharellus* species from South Korea. MycoKeys.

[17] Eyssartier G., Buyck B. (2001). Note nomenclaturale et systématique sur le genre *Cantharellus*. Doc. Mycol..

[18] Buyck B., Henkel T.W., Dentinger B.T.M., Séné O., Hofstetter V. (2016). Multigene sequencing provides a suitable epitype, barcode sequences and a precise systematic position for the enigmatic, *African Cantharellus miniatescens*. Cryptog. Mycol..

[19] Wu X.L., Dai Y.C., Li T.H., Yang Z.L., Song B. (2011). Fungi of Tropical China.

[20] Shao S.C., Tian X.F., Liu P.G. (2012). Two species with intercontinental disjunct distribution of the genus *Cantharellus*. J. Yunnan Agric. Univ..

[21] Yang Z.L., Wu G., Li Y.C., Wang X.H., Cai Q. (2021). Common Edible and Poisonous Mushrooms of Southwestern China.

[22] Shao S.C., Buyck B., Tian X.F., Liu P.G., Geng Y.H. (2016). *Cantharellus phloginus*, a new pink-colored species from southwestern China. Mycoscience.

[23] Kornerup A., Wanscher J.H. (1981). Taschenlexikon der Farben.

[24] Vilgalys R., Hester M. (1990). Rapid genetic identification and mapping of enzymatically amplified ribosomal DNA from several *Cryptococcus* species. J. Bacteriol..

[25] Morehouse E.A., James T.Y., Ganley A.R.D., Vilgalys R., Berger L., Murphy P.J., Longcore J.E. (2003). Multilocus sequence typing suggests the chytrid pathogen of amphibians is a recently emerged clone. Mol. Ecol..

[26] Katoh K., Rozewicki J., Yamada K.D. (2019). MAFFT online service: Multiple sequence alignment, interactive sequence choice and visualization. Brief Bioinform..

[27] Tamura K., Stecher G., Peterson D., Filipski A., Kumar S. (2013). MEGA6: Molecular Evolutionary Genetics Analysis version 6.0. Mol. Biol. Evol..

[28] Stamatakis A. (2006). RAxML-VI-HPC: Maximum likelihood-based phylogenetic analyses with thousands of taxa and mixed models. Bioinformatics.

[29] Ronquist F., Teslenko M., van der Mark P., Ayres D.L., Darling A., Höhna S., Larget B., Liu L., Suchard M.A., Huelsenbeck J.P. (2012). MrBayes 3.2: Effificient bayesian phylogenetic inference and model choice across a large model space. Syst. Biol..

[30] Lanfear R., Frandsen P.B., Wright A.M., Senfeld T., Calcott B. (2017). PartitionFinder 2: New methods for selecting partitioned models of evolution for molecular and morphological phylogenetic analyses. Mol. Biol. Evol..

[31] Moncalvo J.M., Nilsson R.H., Koster B., Dunham S.M., Bernauer T., Matheny P.B., McLenon T., Margaritescu S., Weiß M., Garnica S. (2006). The cantharelloid clade: Dealing with incongruent gene trees and phylogenetic reconstruction methods. Mycologia.

[32] Buyck B., Cruaud C., Couloux A., Hofstetter V. (2011). *Cantharellus texensis* sp. nov. from Texas, a Southern lookalike of *C. cinnabarinus* revealed by tef-1 sequence data. Mycologia.

[33] Buyck B., Moreau P.-A., Courtecuisse R., Kong A., Roy M., Hofstetter V. (2016). *Cantharellus coccolobae* sp. nov. and *Cantharellus garnieri* two tropical members of *Cantharellus* subg. *Cinnabarinus*. Cryptog. Mycol..

[34] Buyck B. (2016). Special issue: *Cantharellus*. Cryptog. Mycol..

[35] Suhara H., Kurogi S. (2015). *Cantharellus cyphelloides* (Cantharellales), a new and unusual species from a Japanese evergreen broad-leaved forest. Mycol. Prog..

[36] Olariaga I., Moreno G., Manjón J.L., Salcedo I., Hofstetter V., Rodríguez D., Buyck B. (2017). *Cantharellus* (Cantharellales, Basidiomycota) revisited in Europe through a multigene phylogeny. Fungal Divers..

[37] Ducousso M., Contesto C., Cossegal M., Prin Y., Rigault F., Eyssartier G. (2004). *Cantharellus garnierii* sp. nov. from nickel mine maquis in New Caledonia. Cryptog. Mycol..

[38] Buyck B., Hofstetter V. (2011). The contribution of tef-1 sequences to species delimitation in the *Cantharellus cibarius* complex in the southeastern USA. Fungal Divers..

[39] Buyck B., Ndolo Ebika S.T., De Kesel A., Hofstetter V. (2020). Tropical African Cantharellus Adans.: Fr. (Hydnaceae, Cantharellales) with lilac-purplish tinges revisited. Cryptog. Mycol..

[40] Feibelman T.P., Bennett J.W., Cibula W.G. (1996). *Cantharellus tabernensis*: A new species from the Southeastern United States. Mycologia.

[41] Ryvarden L., Petersen R. (1971). Notes on cantharelloid fungi IV. Two new species of *Cantharellus*. Sven. Bot. Tidskr..

[42] Buyck B., Lewis D.P., Eyssartier G., Hofstetter V. (2010). *Cantharellus quercophilus* sp. nov. and its comparison to other small, yellow or brown American chanterelles. Cryptog. Mycol..

[43] Petersen R.H. (1985). *Notes on Clavarioid Fungi. XIX*. Colored illustrations of selected taxa, with comments on *Cantharellus*. Nova Hedwig..

[44] Kuo M. (2015). *Cantharellus* *persicinus*. http://www.mushroomexpert.com/cantharellus_persicinus.html.

[45] Bigelow H.E. (1978). The cantharelloid fungi of New England and adjacent areas. Mycologia.

[46] Montoya L., Herrera M., Bandala V.M., Ramos A. (2021). Two new species and a new record of yellow *Cantharellus* from tropical Quercus forests in eastern Mexico with the proposal of a new name for the replacement of *Craterellus confluens*. MycoKeys.

